# The Role of Hydrogen Sulfide in iNOS and APP Localization and Expression in Neurons and Glial Cells Under Traumatic Effects: An Experimental Study with Bioinformatics Analysis and Biomodeling

**DOI:** 10.3390/ijms252211892

**Published:** 2024-11-05

**Authors:** Stanislav Rodkin, Chizaram Nwosu, Evgeniya Kirichenko

**Affiliations:** Research Laboratory "Medical Digital Images Based on the Basic Model", Department of Bioengineering, Faculty of Bioengineering and Veterinary Medicine, Don State Technical University, Rostov-on-Don 344000, Russia

**Keywords:** traumatic brain injury, axotomy, hydrogen sulfide, iNOS, APP, neuron apoptosis, glial cells

## Abstract

Hydrogen sulfide (H_2_S) donors are emerging as promising candidates for neuroprotective agents. However, H_2_S-dependent neuroprotective mechanisms are not yet fully understood. We have demonstrated that an H_2_S donor (sodium sulfide, Na_2_S) reduces the expression of inducible NO synthase (iNOS) and amyloid-beta precursor protein (APP) in damaged neural tissue at 24 h and 7 days following traumatic brain injury (TBI). The application of aminooxyacetic acid (AOAA), an inhibitor of cystathionine β-synthase (CBS), produced the opposite effect. Seven days after TBI, iNOS expression was observed not only in the cytoplasm but also in some neuronal nuclei, while APP was exclusively localized in the cytoplasm and axons of damaged neurons. It was also shown that iNOS and APP were present in the cytoplasm of mechanoreceptor neurons (MRNs) in the crayfish, in axons, as well as in certain glial cells 8 h after axotomy. Na_2_S and AOAA had opposing effects on axotomized MRNs and ganglia in the ventral nerve cord (VNC). Multiple sequence alignments revealed a high degree of identity among iNOS and APP amino acid residues in various vertebrate and invertebrate species. In the final stage of this study, biomodeling identified unique binding sites for H_2_S, hydrosulfide anion (HS^−^), and thiosulfate (S_2_O_3_^2−^) with iNOS and APP.

## 1. Introduction

Neurotrauma is a global healthcare issue, causing severe consequences, including disability and death. Traumatic brain injury (TBI) occupies a unique position among neurotraumas, leading to the death of millions of people annually and significantly reducing the quality of life for survivors. Unfortunately, no clinically effective neuroprotective drugs have been developed or passed clinical trials to protect damaged nervous tissue after TBI. This exacerbates the situation and forces laboratories and pharmaceutical companies worldwide to study the signaling mechanisms underlying the survival of neurons and glial cells in response to mechanical damage and to identify new potential molecular agents with neuroprotective effects [1,2].

H_2_S is a signaling gaseous molecule, part of the classical triad of gasotransmitters alongside nitric oxide (NO) and carbon monoxide (CO). It performs a wide range of functions in the body, from simple antioxidant effects to complex signaling mechanisms of neurotransmission. H_2_S is particularly abundant in the brain, where its synthesis is mainly mediated by the enzyme cystathionine-β-synthase (CBS). Increasingly, H_2_S is drawing attention as a neuroprotective agent due to its pronounced effects on the protection of neurons and glial cells under various pathological conditions, including neurotrauma. However, many H_2_S-dependent signaling mechanisms, especially in the context of TBI, remain unexplored or require further investigation [3,4]. A detailed understanding of how H_2_S exerts its effects at the molecular level could be key to developing clinically effective neuroprotectors.

One of the critical molecular mechanisms in TBI is the H_2_S-associated regulation of apoptotic signaling. Apoptosis is known to be the leading type of cell death in the penumbra zone during TBI [5]. In this pathological process, inducible nitric oxide synthase (iNOS), a key enzyme capable of generating high concentrations of NO in response to stress, plays a crucial role. TBI typically leads to marked iNOS expression, contributing to a series of negative consequences, including enhanced apoptosis.

Thus, in TBI, microglia and astrocytes are activated, leading to increased production of pro-inflammatory cytokines that stimulate iNOS expression. Elevated iNOS activity in such pathophysiological conditions causes a sharp increase in NO concentration, which enhances the inflammatory process. This oxidative impact on surrounding tissues becomes one of the mechanisms that leads to damage to neurons and glial cells. High levels of NO produced by iNOS can interact with superoxide (O_2_^−^) to form peroxynitrite (ONOO^−^), a potent oxidative compound. ONOO^−^ can oxidize lipids, proteins, and DNA, causing damage to cell membranes, structural proteins, and more. This cellular damage promotes apoptosis and necrosis in the injured tissue, enlarging the brain injury area.

At high concentrations, NO stimulates pro-apoptotic mechanisms, including caspase activation, and causes mitochondrial dysfunction. Additionally, iNOS can induce vasodilation and increase vascular wall permeability, which contributes to brain edema and disruption of the blood-brain barrier in TBI. Increased vascular permeability allows toxic substances and immune cells to penetrate the brain, aggravating tissue damage.

H_2_S can block the effects of high NO concentrations through its pronounced reducing properties and by activating several cytoprotective signaling mechanisms [6]. However, the role of H_2_S, particularly a rapid H_2_S donor like Na_2_S, in regulating iNOS localization and expression in brain tissue during TBI has not been previously studied. Additionally, a comprehensive understanding of this process requires an examination of the evolutionary pathway of H_2_S-dependent signaling, which we addressed in our study.

Another protein actively involved in TBI is the amyloid-beta precursor protein (APP), widely known in the context of Alzheimer’s disease pathogenesis. However, its role is much broader and not limited to this pathological condition. APP is involved in many processes, from neuronal differentiation and functioning to neuronal death [7,8]. It is known that APP levels rapidly increase in brain tissue following TBI, and it has been used as a marker of neuronal and axonal damage [9,10,11]. Some studies have shown that H_2_S can regulate this protein under various pathological conditions [12]. For example, the interaction of H_2_S with APP may limit its accumulation in damaged neurons, preventing the formation of neurotoxic fragments. Furthermore, through persulfidation, H_2_S can modify cysteine residues in APP, affecting its stabilization and degradation. However, the role of H_2_S in regulating APP during TBI has been scarcely studied. Given that APP is an evolutionarily conserved protein, its study in both vertebrate and invertebrate nervous tissue is of particular interest.

The interaction of H_2_S with iNOS and APP is directly related to key mechanisms associated with TBI, as both proteins play essential roles in pathophysiological processes such as inflammation, cell death, and oxidative stress. These processes often lead to irreversible damage to neurons and glial cells, intensifying the progression of traumatic changes and laying the foundation for long-term cognitive and motor impairments.

Studying the mechanisms of H_2_S interaction with iNOS and APP opens up new approaches to neuroprotection. A deep understanding of H_2_S-dependent regulation of these proteins could aid in developing new strategies aimed at reducing cell death, inflammation, and oxidative stress in TBI. The use of H_2_S donors, such as sodium sulfide (Na_2_S), may become an effective therapeutic strategy aimed at lowering iNOS activity and toxic APP fragment levels. Combined approaches using inhibitors of enzymes responsible for endogenous H_2_S synthesis can help control H_2_S levels depending on the stage of injury and related inflammation intensity, enabling more precise regulation of detrimental molecular-cellular events in TBI. Research into H_2_S interactions with iNOS and APP will allow for the creation of molecularly targeted agents that interact highly selectively with specific cellular components responsible for inflammation, oxidative stress, and cell death, providing more targeted effects on specific molecular-cellular targets.

**Figure 1:**
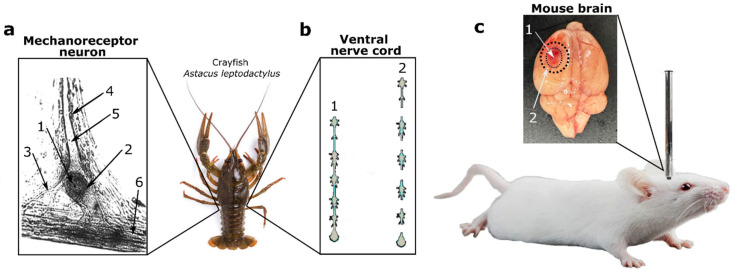
Models of traumatic brain injury and axotomy. (**a**) Crayfish MRN axotomy model: 1—MRN nucleus; 2—MRN cytoplasm; 3—dendrites; 4—satellite glial cells; 5—axon; 6—receptor muscle. (**b**) Crayfish VNC axotomy model: 1—VNC consists of six ganglia interconnected by connectives, which were cut with ophthalmic scissors; 2—axotomized ganglia. (**c**) TBI model induced by falling weight: 1—necrotic zone; 2—penumbra zone under study.

In our study, we investigated the role of H_2_S in the expression and localization of iNOS and APP in the nervous tissue of vertebrate and invertebrate animals under mechanical injury conditions. We used the following H_2_S level modulators: a rapid H_2_S donor (sodium sulfide, Na_2_S) [13,14] and an inhibitor (aminooxyacetic acid, AOAA), which blocks CBS, a key enzyme in H_2_S synthesis [15,16]. The experimental animals selected were mice and freshwater crayfish, *Astacus leptodactylus*, in which three neurotrauma models, including TBI and axotomy, were implemented (Figure 1). The TBI model was induced by dropping a metal rod onto the parietal cortex of the mouse brain (Figure 1c). It is important to note that axotomy is an inevitable consequence of TBI, especially diffuse TBI [17], which is why we used the mechanoreceptor neuron (MRN) of the crayfish (Figure 1a) and its axotomized ventral nerve cord (VNC) (Figure 1b). This also allowed us to trace the evolutionary pathway of H_2_S-dependent regulation of iNOS and APP in injured neurons and glial cells.

The final step of our study was to assess the structural conservation of iNOS and APP in invertebrate and vertebrate animals using bioinformatic analysis and public databases, followed by biomodeling of the interactions between H_2_S, HS^−^, and S_2_O_3_^2−^ with iNOS and APP, along with the visualization of the obtained data.

Thus, in our study, we employed the following models and methods: the TBI model in mice, axotomy models of MRN and VNC, fluorescence microscopy, inhibitor–activator analysis, immunoblotting, bioinformatic analysis, and biomodeling. The use of three models in vertebrate and invertebrate animals allowed for a comprehensive study of the role of H_2_S in regulating the localization and expression of iNOS and APP in neurons and glial cells under traumatic nerve tissue injury, providing an evolutionary perspective on this process. The obtained data have both fundamental and practical significance and may contribute to the development of effective neuroprotective drugs.

## 2. Results

### 2.1. The Role of H_2_S in Regulating iNOS Levels in the Nucleoplasm and Cytoplasm of Damaged Neurons and Astrocytes in the Brain After TBI

It is currently known that all isoforms of NOS can translocate to the nucleus of cells under various pathological conditions [18,19]. However, this process is not fully understood, and the signaling mechanisms underlying it require further investigation. One such mechanism may involve H_2_S, which has particularly high concentrations in the brain and acts as a key molecular player in regulating numerous signaling pathways, including iNOS-mediated mechanisms, under stress conditions. However, the regulation of iNOS by H_2_S has not been thoroughly studied, especially using a donor of H_2_S such as Na_2_S, which has primarily been applied in studies of cardiovascular pathologies [13,14]. We used AOAA as its antagonist—a classical inhibitor of CBS, the enzyme responsible for H_2_S synthesis [15,16].

**Figure 2:**
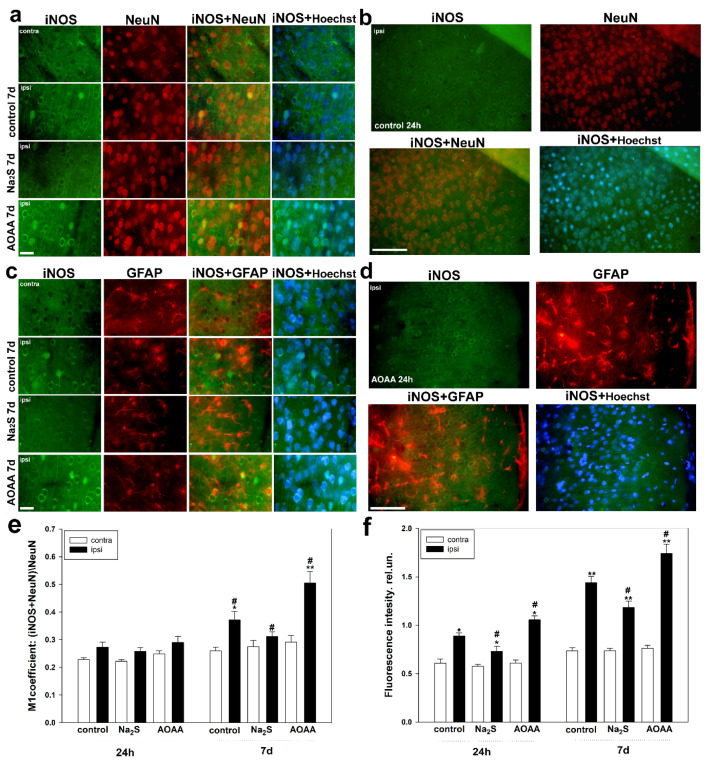
Immunofluorescence microscopy: (**a**) Expression of iNOS (green fluorescence) in the brain cortex of control and experimental groups of animals 7 days after TBI. Scale bar 20 µm. (**b**) Expression of iNOS (green fluorescence) in the brain cortex of the control group 24 h after TBI. Scale bar 50 µm. (**c**) Expression of iNOS (green fluorescence) in the brain cortex of control and experimental groups of animals 7 days after TBI. Scale bar 20 µm. (**d**) Expression of iNOS (green fluorescence) in the brain cortex of the group treated with AOAA 24 h after TBI. Scale bar 50 µm. NeuN—neuronal nuclear marker (red fluorescence); NeuN + iNOS and Hoechst + iNOS—overlay. GFAP—astrocyte marker (red fluorescence); GFAP + iNOS and Hoechst + iNOS—overlay. Hoechst 33342 (blue fluorescence) visualizes the nuclei of all cells. (**e**) Graph showing the M1 coefficient of co-localization of iNOS and NeuN in the contralateral and ipsilateral cortices of the control and experimental groups 24 h and 7 days after TBI. (**f**) Dependence of the average intensity of iNOS fluorescence in the cytoplasm of neurons in the contralateral and ipsilateral cortices of the control and experimental groups 24 h and 7 days after TBI. Contra, contralateral cortex; ipsi, ipsilateral cortex. One-way ANOVA. M ± SEM. *n* = 6. * *p* < 0.05, ** *p* < 0.01—ipsilateral cortex relative to the contralateral cortex of the same animal; # *p* < 0.05—ipsilateral cortex of the experimental group compared to the ipsilateral cortex of the control group within the same time period after injury.

As a result, we were able to demonstrate that the initial level of iNOS is extremely low in both the control and experimental groups in the uninjured cortex of the brain. Isolated neurons were identified in which the cytoplasm exhibited fluorescence of antibodies against iNOS. However, TBI led to an increase in the level of iNOS in neurons and glial cells, as confirmed by the co-localization of iNOS with the neuronal nucleus marker NeuN and astrocytes GFAP (Figure 2a–d). Notably, 24 h after the injury, iNOS was primarily localized in the cytoplasm of neurons, not in the nucleoplasm (Figure 2b). An increase in the level of anti-iNOS fluorescence in the cytoplasm of damaged neurons in experimental groups was observed compared to the nerve cells in the penumbra of the control group. It was established that the expression of iNOS decreased by 17% (*p* < 0.05) with the use of Na_2_S but increased by 19% (*p* < 0.05) relative to control when AOAA was administered 24 h after TBI (Figure 2f). Furthermore, it is noteworthy that iNOS was also detected in damaged axons (Figure 2a,b).

Significant changes in iNOS levels were observed 7 days after TBI. It is important to note that iNOS expression increased not only in the cytoplasm but also in the nucleoplasm of some damaged neurons, as well as in their axons (Figure 2a). At the same time, the level of iNOS in the contralateral uninjured cortex of the mice remained low and was approximately equal to the values recorded 24 h after TBI. Co-localization analysis of iNOS with the nuclear neuronal marker NeuN showed that the level of this enzyme increased by 35% (*p* < 0.05) in the nuclei of nerve cells in the penumbra of the control group 7 days after the injury. Injections of Na_2_S blocked the nuclear translocation of iNOS (*p* < 0.05). Meanwhile, the administration of AOAA led to an increase in the M1 coefficient relative to the ipsilateral cortex of the control group by 33% (*p* < 0.05) (Figure 2e).

**Figure 3:**
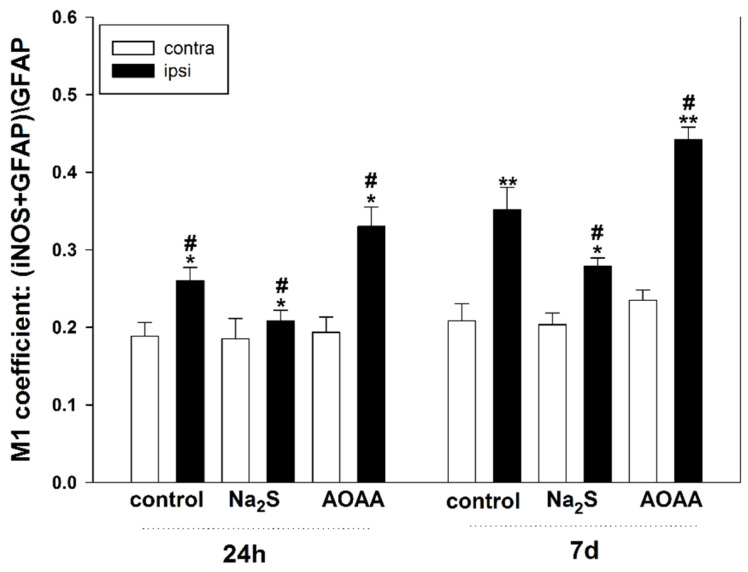
The M1 coefficient of co-localization of iNOS and the astrocyte marker GFAP in the contralateral and ipsilateral cortices of the control and experimental groups 24 h and 7 days after TBI. Contra, contralateral cortex; ipsi, ipsilateral cortex. One-way ANOVA. M ± SEM. *n* = 6. * *p* < 0.05, ** *p* < 0.01—ipsilateral cortex relative to the contralateral cortex of the same animal; # *p* < 0.05—ipsilateral cortex of the experimental group compared to the ipsilateral cortex of the control group within the same time period after injury.

Next, we assessed the expression of iNOS in astrocytes. To visualize them, we used the classical marker GFAP (Figure 2c,d). We established that iNOS is expressed in astrocytes 24 h and 7 days after TBI. The level of iNOS in astrocytes in the uninjured cortex of all groups was hardly detectable. Additionally, the H_2_S modulators used in our study had opposing effects on the level of iNOS in glial cells under traumatic conditions. It was shown that the M1 coefficient decreased in damaged glial cells with the use of Na_2_S relative to the ipsilateral control cortex both 24 h and 7 days after TBI by 26% (*p* < 0.05) and 22% (*p* < 0.05), respectively. However, injections of AOAA resulted in an increased level of iNOS in the penumbra astrocytes compared to the injured glial cells of the control group by 26% (*p* < 0.05) and 27% (*p* < 0.05) at 24 h and 7 days after TBI, respectively (Figure 3).

**Figure 4:**
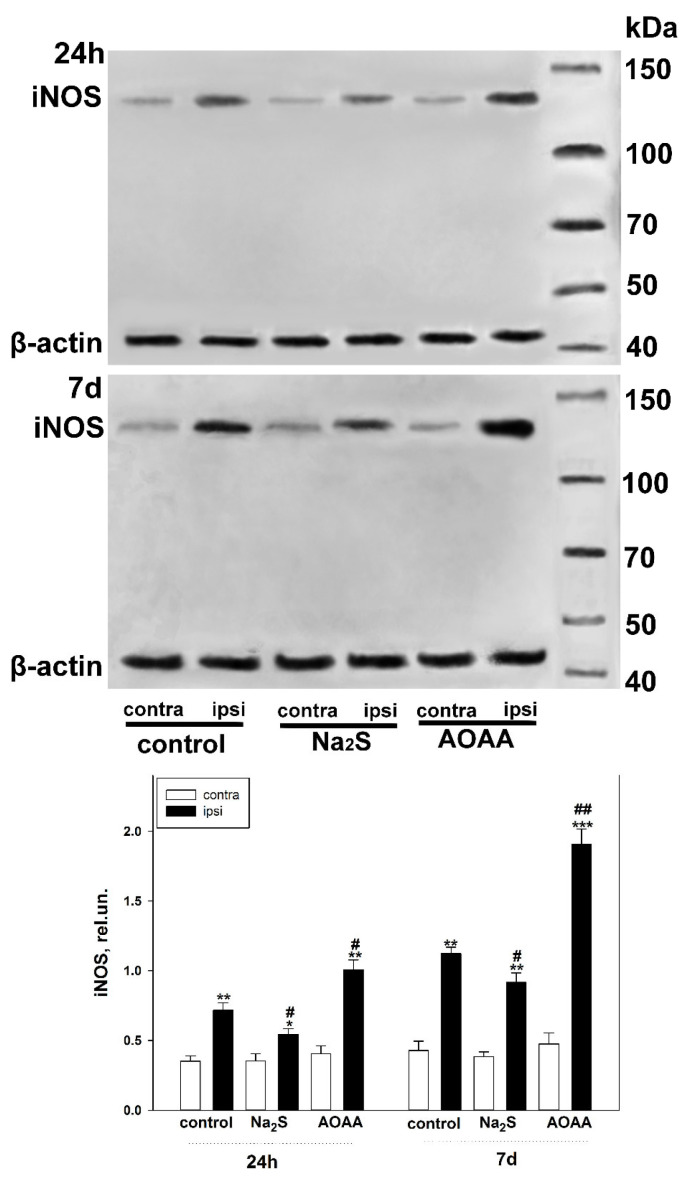
Western blot analysis of iNOS levels in the total brain fraction obtained from control and experimental groups 24 h and 7 days after TBI. One-way ANOVA. M ± SEM. *n* = 6. * *p* < 0.05, ** *p* < 0.01, *** *p* < 0.01—ipsilateral cortex relative to the contralateral cortex of the same animal; # *p* < 0.05, ## *p* < 0.01—ipsilateral cortex of the experimental group compared to the ipsilateral cortex of the control group within the same time period after injury.

We conducted a Western blot analysis to confirm the data from the fluorescent microscopy. The total fraction of brain tissue was used for the analysis. It was found that iNOS increased in the damaged brain cells of both the control and experimental groups 24 h and 7 days after TBI. The level of iNOS was lower in the fraction obtained from the injured area of the brain of mice treated with Na_2_S compared to the control fraction of the ipsilateral hemisphere by 24% (*p* < 0.05) and 20% (*p* < 0.05) at 24 h and 7 days after TBI, respectively. At the same time, the CBS inhibitor had the opposite effect (Figure 4).

### 2.2. Role of H_2_S in Regulating APP Levels in the Cytoplasm of Injured Neurons and Astrocytes in the Brain After TBI

The H_2_S-dependent regulation of APP in the context of TBI is of particular interest. There is a notable lack of research on this topic, making it especially relevant. APP is a well-known protein often discussed in the context of Alzheimer’s disease, but it is also responsible for many functions in the body under normal and pathological conditions, including TBI [9,10,11]. We found that Na_2_S, a rapid H_2_S donor, and its antagonist AOAA, a selective inhibitor of a key enzyme involved in the synthesis of this gasotransmitter, can regulate the accumulation of APP in the cytoplasm of injured neurons and astrocytes, with opposing effects.

**Figure 5:**
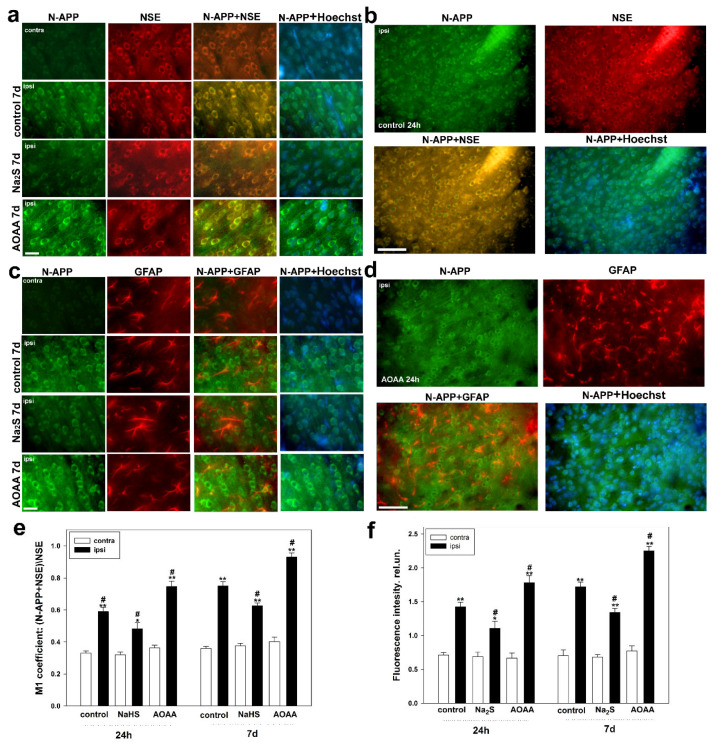
Immunofluorescent microscopy: (**a**) Expression of N-APP (green fluorescence) in the cerebral cortex of control and experimental groups of animals 7 days after TBI. Scale bar 20 μm. (**b**) Expression of N-APP (green fluorescence) in the cerebral cortex of the control group 24 h after TBI. Scale bar 50 μm. (**c**) Expression of N-APP (green fluorescence) in the cerebral cortex of control and experimental groups of animals 7 days after TBI. Scale bar 20 μm. (**d**) Expression of N-APP (green fluorescence) in the cerebral cortex of the group administered AOAA 24 h after TBI. Scale bar 50 μm. NeuN—marker for neuronal nuclei (red fluorescence); NSE + N-APP and Hoechst + N-APP—overlay. GFAP—marker for astrocytes (red fluorescence); GFAP + APP and Hoechst + N-APP—overlay. Hoechst 33342 (blue fluorescence), which visualizes the nuclei of all cells. (**e**) Graph of the M1 colocalization coefficient of N-APP and NSE in the contralateral and ipsilateral cortex of control and experimental groups at 24 h and 7 days post-TBI. (**f**) Dependence of the average fluorescence intensity of N-APP in the cytoplasm of neurons in the contralateral and ipsilateral cortex of control and experimental groups at 24 h and 7 days post-TBI. Contra, contralateral cortex; ipsi, ipsilateral cortex. One-way ANOVA. Mean ± SEM. *n* = 6. * *p* < 0.05, ** *p* < 0.01—ipsilateral cortex relative to the contralateral cortex of the same animal; # *p* < 0.05—ipsilateral cortex of the experimental group compared to the ipsilateral cortex of the control group within the same time period after injury.

Initially, APP levels were relatively low. However, TBI quickly induced its accumulation in neurons and glial cells. APP was found to accumulate in the cytoplasm of neurons and their axons, as well as in astrocytes (Figure 5a–d). We used the specific cytoplasmic neuronal marker NSE to accurately determine APP localization and calculate the M1 coefficient. It was shown that the use of Na_2_S led to a decrease in APP expression in injured nerve cells compared to injured neurons in the control group by 18% (*p* < 0.05) and 16% (*p* < 0.05) at 24 h and 7 days after TBI, respectively (Figure 5e). However, administration of AOAA resulted in the opposite effect, manifesting as an increase in APP expression in the ipsilateral hemisphere relative to the control group penumbra by 26% (*p* < 0.05) and 24% (*p* < 0.05) at 24 h and 7 days post-injury, respectively (Figure 5e).

Additionally, we calculated the fluorescence intensity in relative units of anti-APP in the cytoplasm of neurons. A significant trend was observed, showing an H_2_S-dependent reduction of APP levels with the rapid donor and an opposite effect with the CBS inhibitor (Figure 5f).

**Figure 6:**
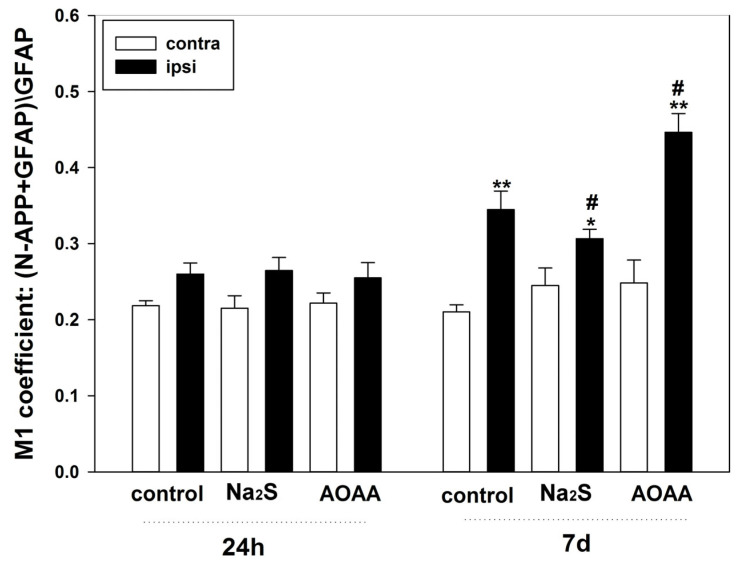
The M1 colocalization coefficient of N-APP and the astrocyte marker GFAP in the contralateral and ipsilateral cortex of control and experimental groups at 24 h and 7 days after TBI. Contra, contralateral cortex; ipsi, ipsilateral cortex. One-way ANOVA. Mean ± SEM. *n* = 6. * *p* < 0.05, ** *p* < 0.01—ipsilateral cortex relative to the contralateral cortex of the same animal; # *p* < 0.05—ipsilateral cortex of the experimental group compared to the ipsilateral cortex of the control group within the same time period after injury.

The next step was to investigate APP expression in astrocytes using the GFAP marker (Figure 5c,d). The results indicated that APP was hardly detectable in healthy glial cells. At 24 h post-TBI, APP expression remained low and did not differ between control and experimental groups. However, at 7 days post-TBI, APP-positive glial cells were found in the injury zone. Their number increased in the AOAA-treated group by 30% (*p* < 0.05) compared to injured astrocytes in the control group. The opposite significant effect (*p* < 0.05) was achieved with Na_2_S administration (Figure 6).

**Figure 7:**
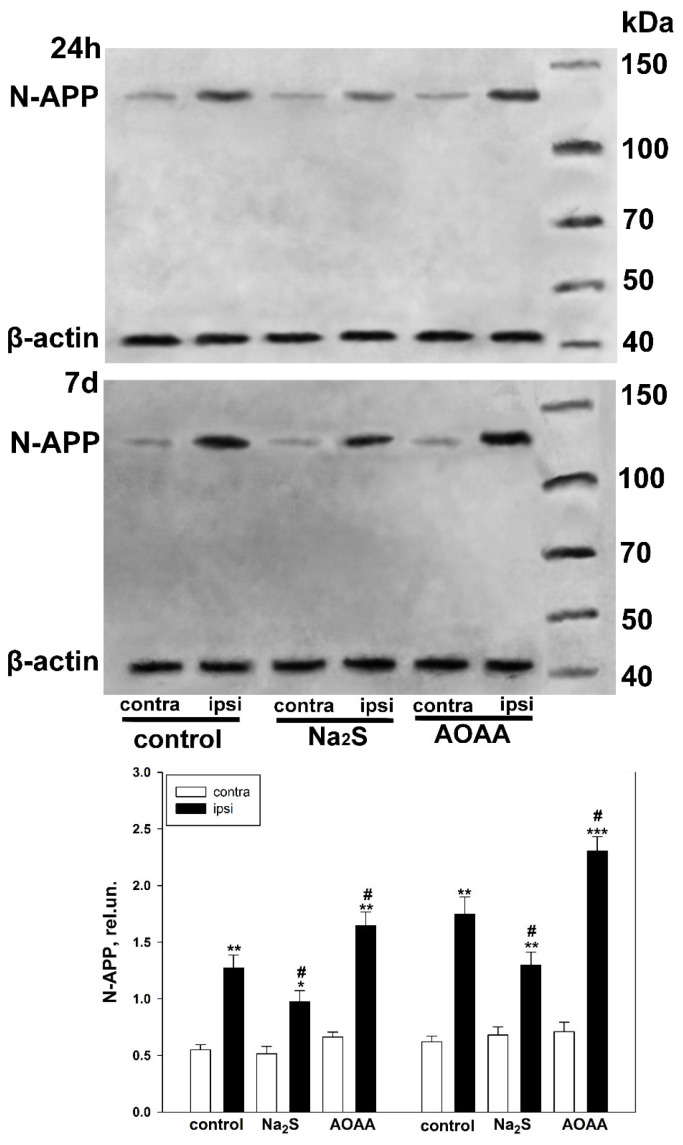
Western blot analysis of N-APP levels in the total brain fraction obtained from control and experimental groups at 24 h and 7 days after TBI. One-way ANOVA. Mean ± SEM. *n* = 6. * *p* < 0.05, ** *p* < 0.01, *** *p* < 0.01—ipsilateral cortex relative to the contralateral cortex of the same animal; # *p* < 0.05—ipsilateral cortex of the experimental group compared to the ipsilateral cortex of the control group within the same time period after injury.

We also conducted a Western blot analysis of the total brain tissue fraction from the control and experimental groups. It was shown that in healthy nerve tissue, the level of APP was low (Figure 7). However, TBI induces APP expression in brain cells, which increases with CBS blockade and decreases with Na_2_S donor administration by 25% (*p* < 0.05) and 29% (*p* < 0.05) compared to the injured nerve tissue of the control group at 24 h and 7 days, respectively (Figure 7).

### 2.3. Role of H_2_S in the Regulation of iNOS Levels in Neurons of the Crayfish Astacus leptodactylus

Now that we have studied the effect of H_2_S on the localization and expression of iNOS and APP in neurons and glial cells of the mouse brain after TBI, there is significant interest in examining these proteins in the context of evolutionary aspects, specifically in the nerve and glial cells of invertebrate animals, as well as investigating H_2_S-dependent signaling mechanisms regulating APP and iNOS in these animals. For this purpose, we selected two neuroglial objects of the river crab, namely the MRN and the VNC. These are convenient model objects for studying neurotrauma, in this case, axotomy, which is an inseparable companion of TBI. It is worth noting that no one has previously studied the effect of the H_2_S donor and CBS inhibitor on these proteins in the river crab under conditions associated with axonal stress.

**Figure 8:**
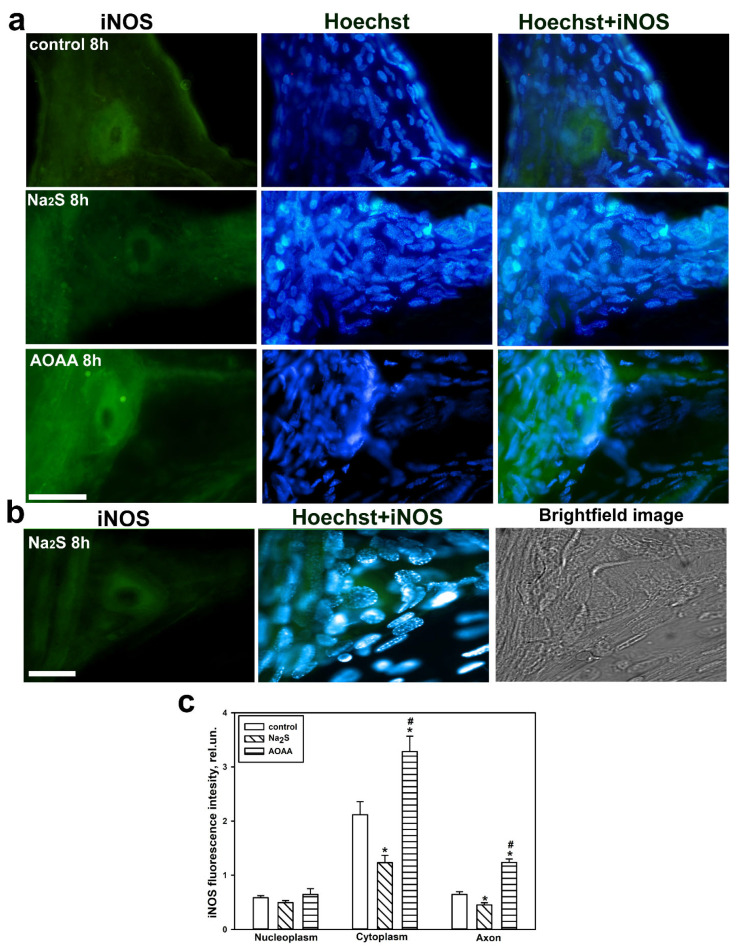
Immunofluorescent microscopy of iNOS localization. (**a**) Expression of iNOS (green fluorescence) in the MRN of the control group and experimental groups eight hours after axotomy. Scale bar: 40 µm. (**b**) Expression of iNOS in the MRN with the addition of Na_2_S eight hours after axotomy. Scale bar: 20 µm. (**c**) Graph of fluorescence intensity of iNOS in the nucleoplasm and cytoplasm of MRN, as well as in the axon eight hours after axotomy. Hoechst + N-APP—overlay. Hoechst—Hoechst 33342 fluorescence, which visualizes the nuclei of all cells, neurons, and glia. One-way ANOVA. Mean ± SEM. *n* = 6. * *p* < 0.05—comparison with a control group; # *p* < 0.05—comparison with the experimental group incubated with Na_2_S.

We demonstrated that in the control MRN, the level of iNOS was primarily localized in the cytoplasm and significantly less in the axon. Additionally, anti-iNOS fluorescence was detected in receptor muscles and very little in glial cells eight hours after axotomy (Figure 8a,b). We did not detect nuclear localization of iNOS in the MRN after axon transection. The introduction of Na_2_S into the chamber where the MRN was isolated reduced the level of iNOS in the cytoplasm by 1.75 times (*p* < 0.05) and 1.5 times in the axon (*p* < 0.05) compared to the control, respectively. The opposite effect was achieved by adding the AOAA inhibitor, which caused a significant increase in iNOS expression in the cytoplasm by 1.5 times (*p* < 0.05) and almost doubled (*p* < 0.05) in the axon of the MRN compared to the control neuroglial preparation, respectively (Figure 8c).

**Figure 9:**
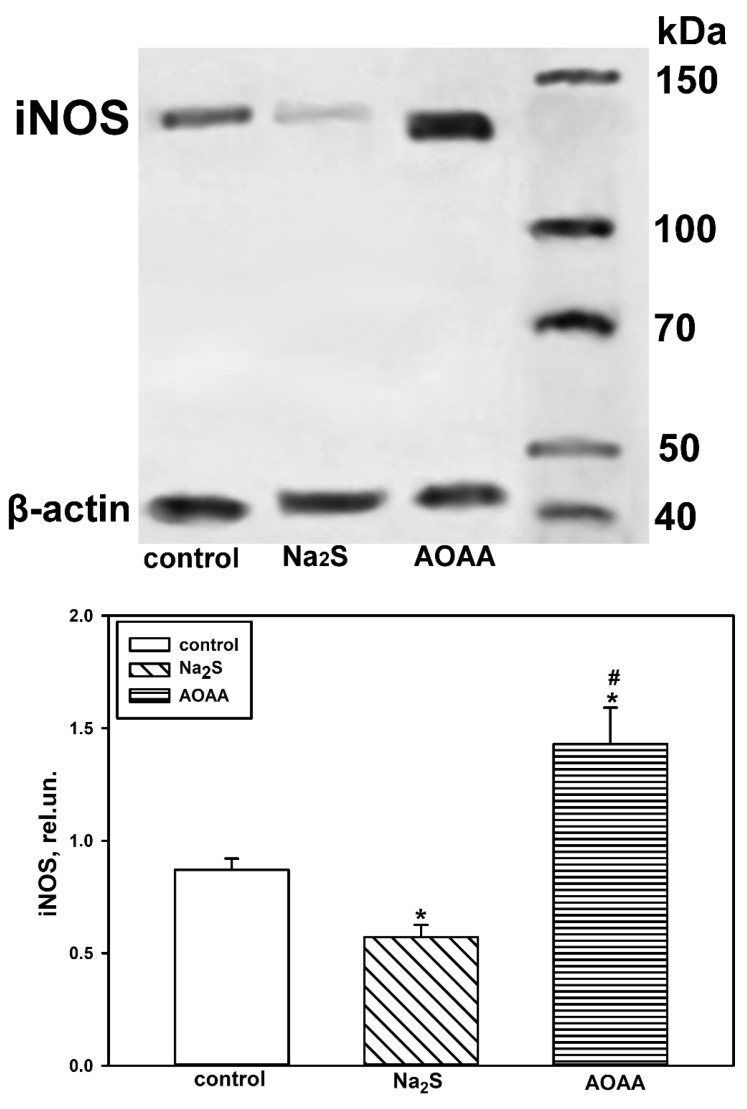
Western Blot. Effect of Na_2_S and AOAA on the expression of iNOS in axotomized ganglia of the ventral nerve cord eight hours after axotomy. One-way ANOVA. Mean ± SEM. *n* = 6. * *p* < 0.05—compared to control, # *p* < 0.05—compared to experimental groups.

Next, we conducted a study on the axotomized ganglia of the VNC in the control and experimental groups. We used Western blot analysis to determine the level of iNOS expression in the total fraction. As a result, we demonstrated that eight hours after axotomy, the level of iNOS was significantly higher than in the group treated with Na_2_S, specifically by 34% (*p* < 0.05), and nearly 1.7 times lower (*p* < 0.05) than in the group of ganglia incubated with AOAA (Figure 9).

### 2.4. Role of H_2_S in the Regulation of APP Levels in Neurons of the Crayfish Astacus leptodactylus

In this part of this study, we aimed to investigate the localization and expression of APP in the nervous tissue of invertebrate animals under conditions of axonal stress, as well as the influence of H_2_S on this process.

**Figure 10:**
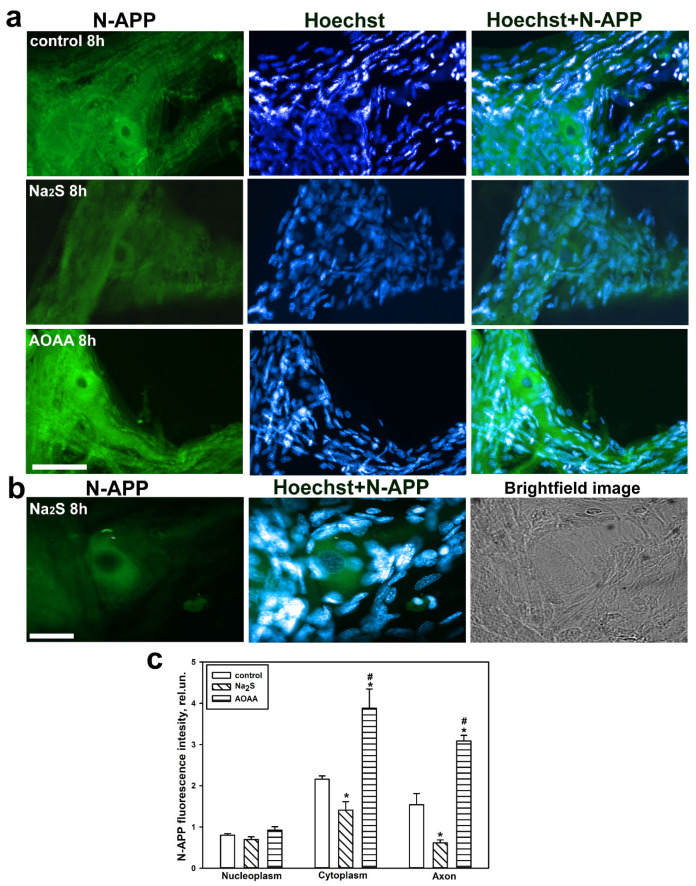
Immunofluorescent microscopy of N-APP localization. (**a**) Expression of N-APP (green fluorescence) in the MRN of the control group and experimental groups eight hours after axotomy. Scale bar: 40 µm. (**b**) Expression of N-APP in the MRN with the addition of Na_2_S eight hours after axotomy. Scale bar: 20 µm. (**c**) Graph of fluorescence intensity of N-APP in the nucleoplasm and cytoplasm of MRN, as well as in the axon eight hours after axotomy. Hoechst + N-APP—overlay. Hoechst—Hoechst 33342 fluorescence, which visualizes the nuclei of all cells, neurons, and glia. One-way ANOVA. Mean ± SEM. *n* = 6. * *p* < 0.05—comparison with a control group; # *p* < 0.05—comparison with the experimental group incubated with Na_2_S.

We demonstrated that APP exhibits a high degree of conserved localization in the cells of vertebrates and invertebrates. In the MRN, APP localized in the cytoplasm and axon, as well as in receptor muscles, and less frequently in glial cells, eight hours after axotomy. The fluorescence of anti-N-APP was not detected in the nucleoplasm (Figure 10a,b). It is worth noting that APP localized along the entire length of the axon up to the site of transection. The use of Na_2_S led to a reduction in APP levels in the cytoplasm of the MRN by 33% (*p* < 0.05) and nearly 2.5 times (*p* < 0.05) in the axon compared to control, eight hours after axotomy, respectively. The opposite effect was observed with the CBS inhibitor (Figure 10c).

**Figure 11:**
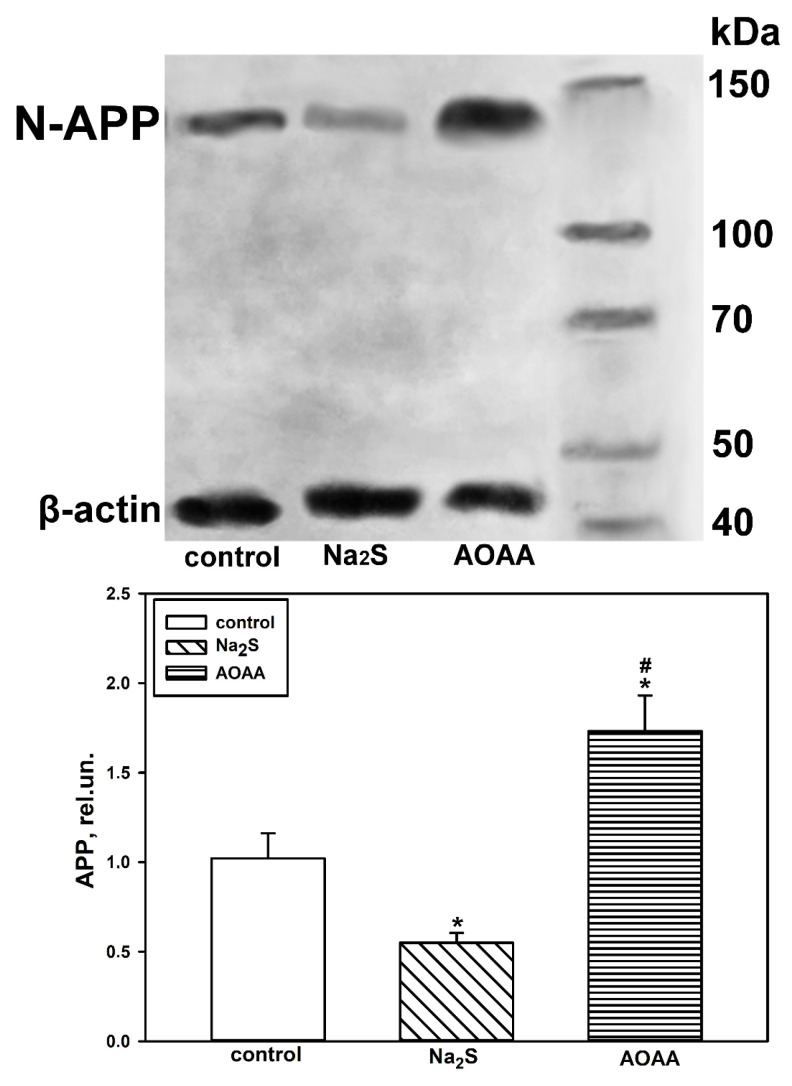
Western Blot. Effect of Na_2_S and AOAA on the expression of N-APP protein in axotomized ganglia of the VNC eight hours after axotomy. One-way ANOVA. Mean ± SEM. *n* = 6. * *p* < 0.05—compared to control, # *p* < 0.05—compared to experimental groups.

Western blot analysis showed that eight hours after axotomy, the H_2_S donor reduced the content of N-APP in the axotomized ganglia by almost half (*p* < 0.05) compared to the control group. The opposite effect was observed when using AOAA (Figure 11).

### 2.5. Analysis of the Evolutionary Conservation of iNOS and APP

We performed multiple alignments of the amino acid residue sequences of iNOS and APP across vertebrates and invertebrates to assess the conservation nature of these proteins across evolution. It was shown that the highest identity values were observed between iNOS of house mouse and human (81.12%). This indicates a strong degree of conservation of iNOS between mammals, which is logical given their evolutionary proximity. Other vertebrates, such as the domestic chicken and rainbow trout, also show relatively high levels of identity with other vertebrates. For example, iNOS in chicken shares 70.48% identity with iNOS in humans. This indicates moderate conservation of the protein among vertebrates (Table 1).

Although trout is more distant from mammals, the identity of its iNOS with mammalian proteins is still maintained at a level of about 58–60%, which suggests that the basic functions and structure of this enzyme remain conserved even in different classes of vertebrates (fish, birds, mammals).

Despite the fact that there are significant differences in the iNOS protein sequences between vertebrates and invertebrates, the identity matrix data still indicate a high affinity between the iNOS of these groups of organisms. This reflects the importance of conserving key functions of the iNOS protein for biological processes such as NO synthesis. Thus, it was shown that the identity of iNOS between a vertebrate organism such as humans (*Homo sapiens*) and an invertebrate such as fruit flies (*Drosophila melanogaster*) is 44.56%, and between humans and shrimp (*Penaeus chinensis*) is 49.34%. Although these values are lower than those between vertebrates, they are still significant given the large evolutionary distance between these organisms. For such distant groups as vertebrates and invertebrates, maintaining an identity of 44–49% indicates the conservation of key functional regions of the iNOS protein. This confirms the high affinity of iNOS between different types of organisms. It is also worth noting that iNOS in invertebrates demonstrates some conservation between themselves. For example, shrimp and *Drosophila* have 56.98% identity, which is higher than their identity with vertebrates (Table 1).

**Figure 12:**
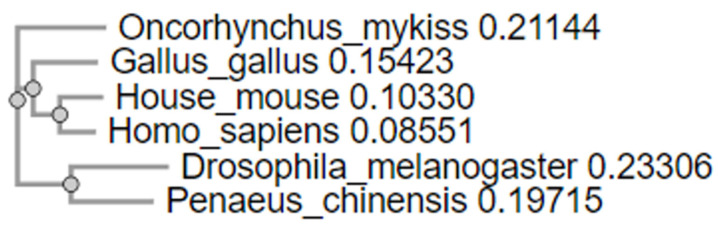
Phylogenetic tree calculated based on multiple comparisons for iNOS from different organisms.

Also, to analyze the evolutionary relationships between proteins, a phylogenetic tree was constructed based on multiple alignments of amino acid sequences performed using the Clustal Omega method. Analysis of the tree allows us to draw conclusions about how iNOS evolved in different lineages. In the case of detection of convergent evolution, it can be assumed that similar functional regions of iNOS responsible for similar functions in different organisms evolved independently of each other (Figure 12).

Interesting results were also obtained when analyzing the amino acid sequences of APP in different organisms. The identity values between *Takifugu rubripes*, *Mus musculus*, and *Homo sapiens* are more than 69% (Table 2). This indicates the presence of homology between the APP proteins in these vertebrates, which confirms their common evolutionary origin and similarity of functions. The high degree of identity between mammals and fish also indicates the importance of APP in the physiology of these species.

*Doryteuthis pealeii* and *Drosophila melanogaster* show an identity of less than 32% (Table 2). Despite this, the presence of certain sequences similar to APP sequences in vertebrates indicates the presence of conserved regions, which may indicate functional analogies in the process of APP regulation. Low identity values between *Drosophila melanogaster* and other species (e.g., 27.98% with *Takifugu rubripes*) may indicate a more distant evolutionary relationship, as well as differences in the functions of APP in these organisms (Table 2). This may be due to adaptations required for different ecosystems and physiological requirements.

At the same time, the presence of identity of about 32% in invertebrates such as *Doryteuthis pealeii* and *Hirondellea gigas* also indicates some homologous aspects between APP proteins in these species (Table 2).

**Figure 13:**
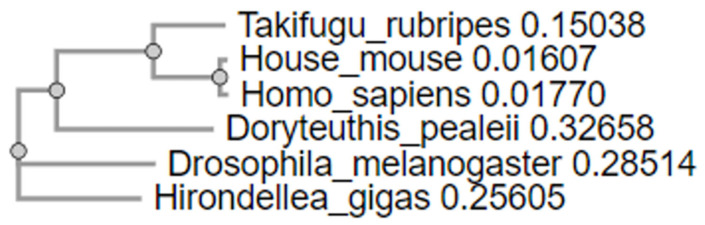
Phylogenetic tree calculated based on multiple comparisons for APP from different organisms.

The phylogenetic tree also allows us to draw conclusions about how APP evolved in different lineages. If convergent evolution is detected, it can be assumed that similar functional regions of APP, responsible for similar functions in different organisms, evolved independently (Figure 13).

### 2.6. Analysis of the Interaction of H_2_S, HS^−^ and S_2_O_3_^2−^ with Functionally Important Regions of iNOS and APP Using Computer Biomodeling

Docking of H_2_S, HS^−^, and S_2_O_3_^2−^ with the iNOS monomer (PDB ID: 1DWW) was carried out in order to clarify the differences in their interaction with this enzyme. We were interested in comparing how the affinity of these compounds would differ. It is known that 80% of H_2_S under physiological conditions exists as HS^−^ and only 20% retains a gaseous form [20]. At the same time, H_2_S can be oxidized to thiosulfate (S_2_O_3_^2−^) and in reactions catalyzed by mitochondrial enzymes [21].

For docking, the UCSF Chimera program was used for protein preparation, and the docking process itself was performed on the SwissDock platform (online service, http://www.swissdock.ch/; accessed on 10 October 2024). To better understand the spatial organization and evaluate the interactions of the three ligands with the studied proteins, 3D visualization of the molecular docking results was performed. This visualization is presented in the figures below using different display formats, which facilitates the analysis of the binding regions and the identification of key interactions stabilizing the ligand–protein complex.

The results yielded calculated affinities for each complex, as well as detailed information on the hydrogen bonds between the ligands and amino acid residues of the iNOS protein. The table shows the affinity values for the models, where the affinity is estimated in kcal/mol. The results were almost the same between H_2_S and HS^−^. However, they were very different from S_2_O_3_^2−^(Table 3).

The highest affinity for iNOS was demonstrated by S_2_O_3_^2−^, with binding energies ranging from −3.465 to −2.666 kcal/mol, indicating a stronger interaction compared to H_2_S and HS^−^, for which the maximum affinity values are around −0.785 kcal/mol and −0.785 kcal/mol, respectively. As the model number increased, the binding affinity gradually decreased for all three ligands, which may indicate differences in their positioning in the iNOS active site (Table 3).

**Figure 14:**
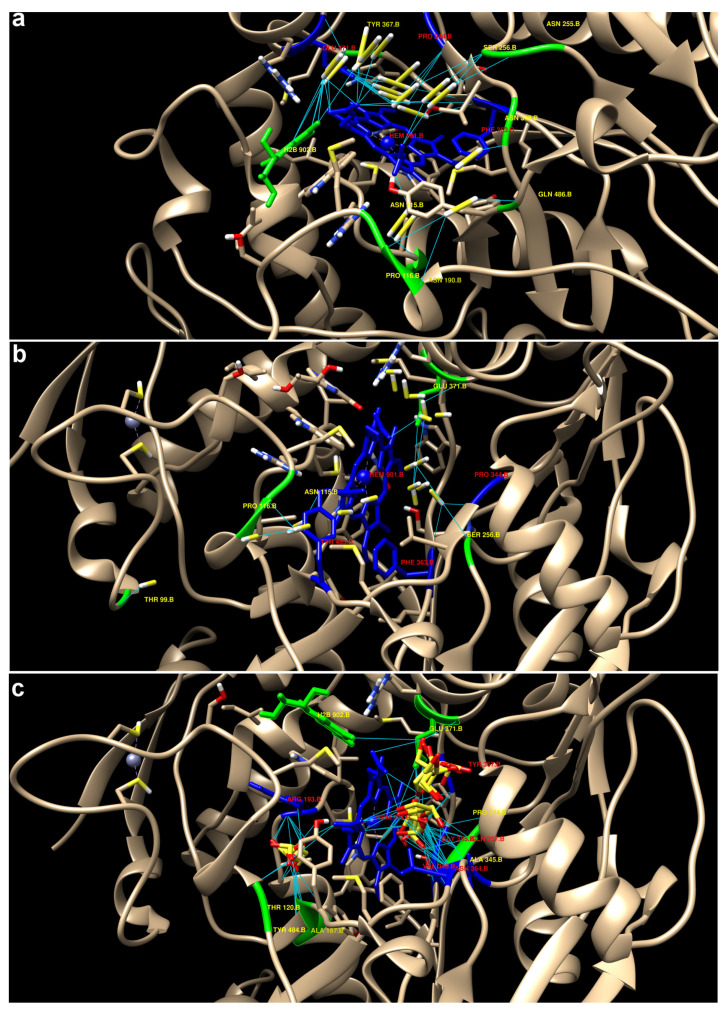
Visualization of the docking results of H_2_S, HS^−^, and S_2_O_3_^2−^ with iNOS monomer: key region. (**a**) Iinteraction of H_2_S with iNOS; (**b**) interaction of HS^−^ with iNOS; (**c**) interaction of S_2_O_3_^2−^ with iNOS. For a better understanding of the binding mechanisms, hydrogen bonds (blue stripes) between H_2_S, HS^−^, and S_2_O_3_^2−^ with iNOS are shown. Regions with the most stable interactions with ligands are highlighted in blue and have red names. Regions with less stable interactions with ligands are highlighted in green and have yellow names.

Hydrogen bonds were calculated to analyze the interactions of ligands with key residues of iNOS. It was shown that H_2_S forms hydrogen bonds with several important amino acid residues, including PRO 344, GLU 371, ASP 376, and the heme group (HEM 901). For example, the S-atom of H_2_S interacts with the oxygen in the side chain of GLU 371 (distances of 3.701 Å and 3.841 Å), as well as with the residue HEM 901 through hydrogen bonds with the atoms O2D and O2A. Interestingly, the interaction with PRO 344 and GLU 371 is the most stable and multiple in different models. The interaction with HEM 901, judging by the significant D. A distance may indicate the potential importance of this bond in the regulation of the enzymatic activity of iNOS. The ability of H_2_S to form hydrogen bonds with important amino acid residues such as PRO 344, GLU 371, ASP 376, and the heme group of HEM 901 indicates its potential stable interaction in the active site (Figure 14).

HS^−^ also forms hydrogen bonds with PRO 344, ASP 376, and GLU 371. Of particular interest is the strong interaction with the residue TYR 485 (distance of 3.884 Å), which may play a role in stabilizing the ligand–protein complex. The interaction with residues ASP 376 and GLU 371 is similar to that observed for H_2_S, but its stability is less pronounced due to the large distances between the hydrogen bond donors and acceptors (Figure 14). HS^−^ forms similar bonds with a number of amino acid residues, which may be important for stabilizing the ligand–protein complex.

The interaction of S_2_O_3_^2−^ with iNOS involves a wide range of hydrogen bonds, covering important residues in the catalytic region and near the heme group (HEM 901). Thus, it was shown that ARG 193 forms hydrogen bonds with the oxygen atoms of the ligand (LIG 1 O) at distances of 3.113 Å, 4.571 Å, 5.575 Å, 3.025 Å, 3.107 Å, and others, indicating an active interaction with this region of the protein. GLN 257 interacts with various oxygen atoms of the ligand at distances of 2.994 Å, 3.046 Å, 3.065 Å, 3.099 Å, and others, confirming stable hydrogen bonding interactions. TRP 340 forms multiple hydrogen bonds, including interactions at distances of 3.831 Å, 4.213 Å, and 5.948 Å. TYR 341 exhibits a range of interactions with the ligand, including distances of 3.161 Å, 3.722 Å, and up to 5.285 Å, indicating a possible role in ligand stabilization. VAL 346, ASN 364, GLY 365, TYR 367, ARG 382, and other residues also participate in hydrogen bonding with various ligand oxygen atoms, demonstrating distributed interactions across iNOS (Table 3). The interaction of S_2_O_3_^2−^ with iNOS covers a broad spectrum of amino acids and exhibits distributed hydrogen bonds throughout the binding pocket, supporting the hypothesis of its stronger binding affinity to iNOS and potential impact on catalytic activity.

**Figure 15:**
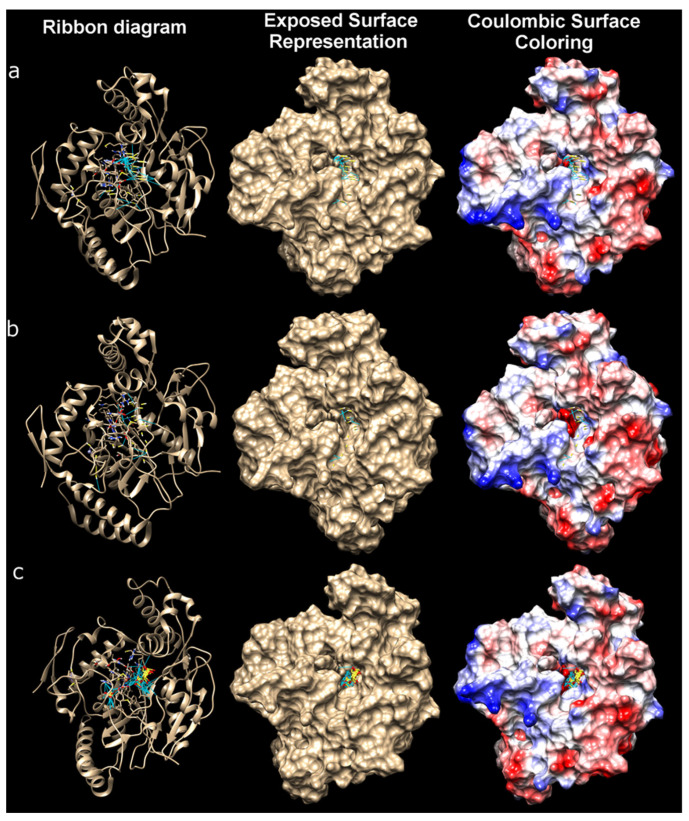
Visualization of the docking results of H_2_S, HS^−^, and S_2_O_3_^2−^ with the iNOS monomer. (**a**) Interaction of H_2_S with iNOS; (**b**) interaction of HS^−^ with iNOS; (**c**) interaction of S_2_O_3_^2−^ with iNOS. Ribbon diagram—three-dimensional schematic images of the protein structure with the ligand; Exposed Surface Representation—representation of the exposed surface showing the sites of interaction of the ligands; Coulombic Surface Coloring—electrostatic map of the iNOS surface showing the distribution of positively (blue) and negatively (red) charged regions around the binding pocket where the ligands are anchored. To better understand the binding mechanisms, hydrogen bonds (blue stripes) between H_2_S, HS^−^, and S_2_O_3_^2−^ with iNOS are shown.

A large-scale visualization of molecular docking to study the interactions of three ligands H_2_S, HS^−^, and S_2_O_3_^2−^ with the iNOS protein monomer is shown in Figure 15. The three-dimensional structures are shown in different visualization formats for a better understanding of the interactions in the binding pocket of the protein.

By analyzing the docking results between the E2 domain of APP and three ligands (H_2_S, HS^−^, and S_2_O_3_^2−^), the following important aspects can be distinguished by dividing them into two components: calculated affinities and hydrogen bonds. This allows us to estimate both the strength of the interactions and the key amino acids involved in the binding. The affinity of H_2_S with the E2 domain of APP varies from −0.701 to −0.391 kcal/mol. The lowest affinity value was −0.701, and the highest was −0.391. This indicates a relatively weak interaction between H_2_S and the E2 domain, which is consistent with its small size and polarity. In turn, the interaction of HS^−^ with the E2 domain of APP varies from −0.700 to −0.390 kcal/mol. These data are almost identical to the results for H_2_S, indicating a similar nature of the interaction, despite the negative charge of HS^−^. For S_2_O_3_^2−^, the affinity varies from −2.636 to −1.745 kcal/mol (Table 4). These values are significantly lower (in absolute value) than those of H_2_S and HS^−^, indicating a much stronger interaction with the E2 domain. This may be due to the more complex structure and polarity of this ligand, which provides additional binding opportunities.

**Figure 16:**
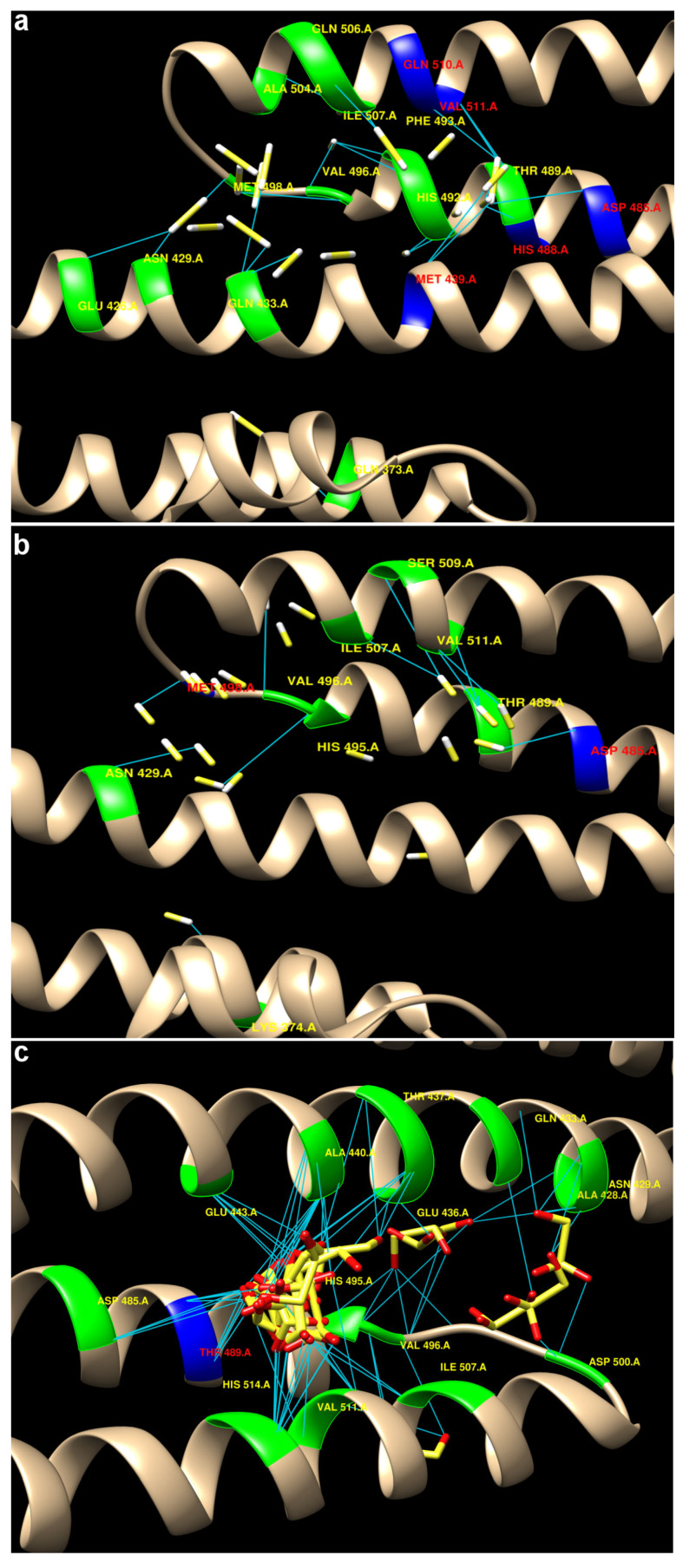
Visualization of the docking results of H_2_S, HS^−^, and S_2_O_3_^2−^ with the E2 domain of the APP protein: the key region. (**a**) Interaction of H_2_S with the E2 domain; (**b**) interaction of HS^−^ with the E2 domain; (**c**) interaction of S_2_O_3_^2−^ with the E2 domain. For a better understanding of the binding mechanisms, hydrogen bonds (blue stripes) between H_2_S, HS^−^, and S_2_O_3_^2−^ with the E2 domain are shown. Regions with the most stable interaction with ligands are highlighted in blue and labeled in red. Regions with less stable interaction with ligands are highlighted in green and labeled in yellow.

H_2_S interactions involve a number of amino acid residues: ASP 485, HIS 488, THR 489, MET 439, GLN 510, VAL 511, and HIS 514. The shortest distances between donors and acceptors are observed for HIS 514 (3.029 Å) and THR 489 (3.159 Å), indicating strong hydrogen bonds with these amino acids. Significant interactions are also observed with GLN 510, ASP 485, and THR 489, indicating an important role for these residues in H_2_S binding (Figure 16).

HS^−^ forms hydrogen bonds with GLU 443, ASP 485, HIS 514, and MET 498, among others. The shortest distance between donors and acceptors is observed for GLU 443 (2.269 Å), indicating a particularly strong interaction with this acidic amino acid. HIS 514 and THR 489 also play an important role in HS^−^ binding, as evidenced by distances less than 4 Å (Figure 16).

In the case of S_2_O_3_^2−^, hydrogen bonds are present, but many of them have quite large distances between donors and acceptors (e.g., GLN 433–4.819 Å, GLU 443–5.165 Å), indicating weak or absent hydrogen bonds. The strongest interactions are observed with THR 489 (distances 3.469–3.975 Å), indicating a key role of this residue in S_2_O_3_^2−^ binding (Figure 16).

**Figure 17:**
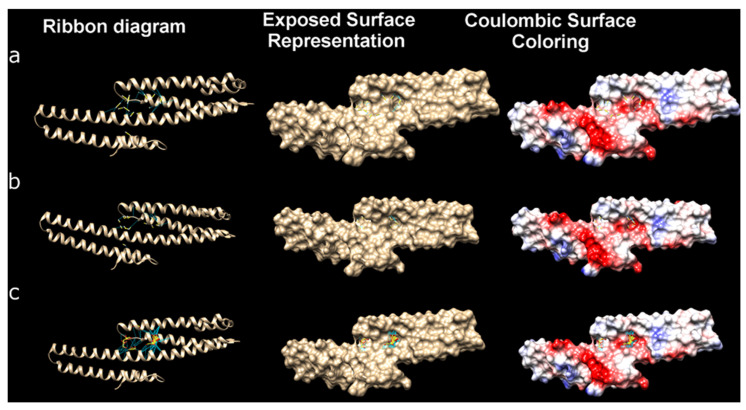
Visualization of the docking results of H_2_S, HS^−^, and S_2_O_3_^2−^ with the E2 domain of the APP protein. (**a**) Interaction of H_2_S with the E2 domain; (**b**) interaction of HS^−^ with the E2 domain; (**c**) interaction of S_2_O_3_^2−^ with the E2 domain. Ribbon diagram—three-dimensional schematic images of the protein structure with the ligand; Exposed Surface Representation—representation of the exposed surface showing the sites of interaction of the ligands; Coulombic Surface Coloring—electrostatic surface map of the E2 domain showing the distribution of positively (blue) and negatively (red) charged regions around the binding pocket where the ligands are anchored. To better understand the binding mechanisms, hydrogen bonds (blue stripes) between H_2_S, HS^−^ and S_2_O_3_^2−^ with the E2 domain are shown.

A large-scale visualization of molecular docking to study the interactions of three ligands H_2_S, HS^−^, and S_2_O_3_^2−^ with the E2 domain of the APP protein is shown in Figure 17. The three-dimensional structures are shown in different visualization formats for a better understanding of the interactions in the binding pocket of the protein.

Thus, the docking results showed that S_2_O_3_^2−^ exhibits the highest affinity for iNOS and the E2 domain of APP among the three studied ligands, suggesting its potentially more stable and robust interaction with these proteins. Strong and distributed hydrogen bonds across the iNOS binding pocket for S_2_O_3_^2−^ may imply this ligand’s involvement in modulating the catalytic activity of iNOS. The observed interactions of HS^−^ and H_2_S, although weaker, also highlight the significance of certain amino acid residues in stabilizing these ligands. Since the results demonstrate that S_2_O_3_^2−^ may play an important role in interactions with iNOS and APP, further investigation into its biological activity is justified.

## 3. Discussion

TBI is the most common type of neurotrauma and poses a serious threat to human health and life. Every year, millions of people worldwide fall victim to TBI, with many either becoming disabled or losing their lives. Even those who recover and return to normal life may later face various negative consequences, such as mental disorders, dementia, and more. To date, numerous proteins are known to play critical roles in TBI. Of particular interest are iNOS and APP, which are key players in TBI pathogenesis. It is known that H_2_S can act as a molecular regulator of these proteins under various pathological conditions [3,4]. However, its role in controlling iNOS and APP levels in neurons and glial cells during TBI has not been extensively studied.

In our study, we demonstrated that H_2_S can regulate iNOS and APP expression in the nervous tissue of both vertebrates and invertebrates. This study was conducted on three model organisms, including the parietal cortex of the mouse brain, as well as the MRNs and VNC of *Astacus leptodactylus*. The obtained neuroglial preparations were analyzed using modern molecular biology methods, followed by bioinformatics analysis to assess the conservation of iNOS and APP across different animal classes. First, we will discuss the results of immunofluorescence microscopy and Western blot analysis regarding vertebrates and invertebrates. We will then conclude with an evaluation of bioinformatics data.

The level of iNOS in healthy brain tissue cells was minimal. It is known that iNOS belongs to the Ca^2^⁺-independent isoform of nitric oxide synthases (NOS), capable of generating high cytotoxic concentrations of NO, unlike neuronal and endothelial NOS. This enzyme is found in many cells, including neurons and glia. iNOS expression typically occurs as a response to stress factors, including mechanical tissue damage. In our previous research, we demonstrated the significant role of iNOS in the expression of the key pro-apoptotic protein p53 and cell death in dorsal root ganglia after sciatic nerve transection [22]. iNOS is one of the key proteins responsible for the death of neurons and glial cells in TBI. In our study, iNOS expression rapidly increased in neurons and glial cells 24 h and 7 days post-TBI. However, in neurons, iNOS was localized in the cytoplasm 24 h after injury. Only after 7 days were nerve cells with nuclear iNOS expression observed, as confirmed by colocalization analysis of iNOS with the nuclear marker NeuN. Reduced iNOS accumulation was observed with the use of an H_2_S donor and was enhanced by CBS inhibition. This aligns with previous studies showing that H_2_S can inhibit iNOS. One potential mechanism for this effect may involve H_2_S-dependent inhibition of the iNOS transcription factor NF–κB [23,24]. However, other studies suggest that H_2_S may induce NO synthesis through the activation of iNOS and NF–κB [25]. Additionally, H_2_S can bind to NO, forming nitroxyl (HNO), which can modify proteins by forming disulfide bonds. For example, HNO can actively modify glutathione (GSH), blocking its antioxidant effects [26]. This effect, however, may be associated with cytotoxic levels of H_2_S.

Significant nuclear iNOS accumulation suggests the enzyme’s translocation to the nucleoplasm of nerve cells during stress reactions. It is known that iNOS can migrate from the cytoplasm to the nucleus under various pathological conditions [18,19]. In our previous studies, we also observed enhanced nuclear localization of iNOS in peripheral nervous system neurons during axotomy [22]. Moreover, we observed iNOS accumulation in the axons of damaged neurons following TBI. Several studies have shown that iNOS can accumulate in Schwann cells [27] or in the axon itself [28]. It is possible that iNOS may have been transported centrifugally into the axon, as was previously demonstrated with nNOS in sensory neurons [29].

The increase in iNOS expression in astrocytes during TBI is consistent with previous studies. It has been shown that cortical astrocytes express iNOS in response to TBI [30,31]. The use of Na_2_S reduced iNOS levels in astrocytes, while the opposite effect was achieved using AOAA. These findings are in line with previous research, which has demonstrated that H_2_S can effectively reduce iNOS expression in this cell type. This effect may occur through an H_2_S-dependent pathway that lowers p38 MAPK levels, the inhibition of which can lead to reduced iNOS expression [32]. The induction of iNOS by AOAA indicates the critical role of CBS in regulating this enzyme. H_2_S can effectively bind to NO and its aggressive free radical products, reducing stress-induced iNOS production in microglia [33]. It has also been reported that exogenous H_2_S can restore CBS expression, which decreases in brain cells during stress responses. However, AOAA blocks this effect, exacerbating apoptosis in damaged neurons and glial cells [20].

We also closely examined iNOS localization in MRNs of *Astacus leptodactylus*. Eight hours after axotomy, iNOS was primarily localized in the cytoplasm and, to a lesser extent, in the axon and was also found in muscle tissue and, to a small degree, in glial cells. Nuclear iNOS localization was not detected after axon transection. H_2_S donors had a pronounced effect on iNOS levels, reducing its expression. However, the use of AOAA led to the opposite effect. It has been reported that in invertebrates, the main sites of NO expression and localization are cells of the central and peripheral nervous systems, as well as muscle tissue [34]. NOS plays a significant role in invertebrates, being responsible for functions such as memory, learning, and neuron migration [35]. In *Pacifastacus leniusculus*, NO has been shown to play an essential role in regulating glial cell apoptosis [36]. Additionally, in our studies, we showed that iNOS is involved in neuroglial interactions during photodynamic therapy in *Astacus leptodactylus* [37].

It is also known that H_2_S is expressed in invertebrates. The use of the AOAA inhibitor almost completely blocked H_2_S production, indicating the significant role of CBS in H_2_S synthesis in this group of organisms [38]. H_2_S is also detected in the nervous system of crustaceans, including freshwater crayfish *Procambarus clarkii* [39].

Next, we studied the expression and localization of APP. Both full-length APP and its proteolytic cleavage products, such as sAPPα, sAPPβ, Aβ, and AICD, exhibit biological activity [40]. We demonstrated that APP is exclusively localized in the cytoplasm and neuronal axons, consistent with recent studies showing that full-length APP in neurons is localized to the cytoplasm and does not enter the nucleus [41,42]. However, there is evidence of interaction and formation of a relatively stable complex of full-length APP that may translocate to the nuclear region. APP can also bind to ganglioside GM1, which is widely present in nervous tissue and localized in the cytoplasm and nuclear envelope [43]. Several authors have demonstrated that APP accumulates in axons during neuronal injury, and it is suggested that APP is actively produced and transported along the axon to areas where intercellular contacts are disrupted [9,10]. We did not observe APP accumulation in the axons of uninjured neurons. These data support previous research showing that APP expression appears only in the axons of damaged nerve cells [44].

TBI-induced APP accumulation in neurons and glial cells. It is known that APP levels rapidly increase after central nervous system injury, and APP is considered a marker of axonal damage [45]. Many studies have shown that APP expression increases in TBI [11]. However, the role of H_2_S in this process has hardly been explored. We were able to show that H_2_S can act as a regulator of APP levels in both neurons and astrocytes, reducing its expression. This mechanism may be due to H_2_S suppressing APP expression by activating antioxidant defense mechanisms: nuclear factor erythroid 2-related factor 2 (Nrf2), heme oxygenase-1 (HO-1), and glutathione S-transferase (GST), among others [46].

We also detected APP in the nervous tissue of the river crayfish *Astacus leptodactylus* after axotomy. APP was localized in the cytoplasm of MPN, its axon, receptor muscles, and, less frequently, glial cells 8 h after axon transection. The use of Na_2_S led to a reduction in APP levels in the cytoplasm and other parts of the MPN. However, the opposite effect was caused by a CBS inhibitor. It is known that invertebrates express APP. It is a conserved protein that performs similar functions in various animal species [47].

Additionally, we performed multiple sequence alignments for the amino acid sequences of iNOS and APP in vertebrates and invertebrates to assess the conservation of these proteins in an evolutionary context. The highest degree of identity for iNOS was observed between *Homo sapiens* and *Mus musculus*, reaching 81.12%, confirming significant conservation of iNOS among mammals, which is expected given their close evolutionary relationship. Moreover, *Gallus gallus* shows 70.48% identity with human iNOS, indicating moderate conservation of the protein among different vertebrate classes, such as birds and mammals.

Interestingly, even more distantly related species, such as *Oncorhynchus mykiss*, maintain substantial identity (58–60%) with mammalian iNOS. This suggests that the enzyme’s essential functions and structure remain stable across various vertebrate classes, likely due to its role in vital processes like NO synthesis.

On the other hand, iNOS sequences between vertebrates and invertebrates show considerable differences. Identity levels between *Drosophila melanogaster* and *Penaeus chinensis* relative to humans are 44.56% and 49.34%, respectively. However, for such distant groups as vertebrates and invertebrates, an identity level of 44–49% suggests the conserved nature of functional iNOS regions. Notably, the identity between invertebrates, such as shrimp and fruit flies, is 56.98%, indicating a relationship in iNOS structure within this animal group.

Analysis of APP also yielded interesting results. Identity between *Takifugu rubripes*, *Mus musculus*, and *Homo sapiens* exceeds 69%, indicating homology among the APP proteins in these vertebrates. This supports shared evolutionary roots and functional similarity of the APP protein across species, emphasizing its importance in physiological processes. It is known that APP belongs to a family of conserved transmembrane proteins with a long evolutionary history [48]. However, in invertebrates such as *Doryteuthis pealeii* and *Drosophila melanogaster*, the identity is less than 32%. Despite this, conserved regions similar to vertebrate APP are observed, which may indicate functional analogies in APP regulation despite the evolutionary distance. Many studies demonstrate that despite evolutionary divergence in amino acid sequences across species, APP retains key functions such as cell adhesion, neuronal migration, and synaptogenesis [48]. It is also noteworthy that the genomes of invertebrates encode a homolog of APP, referred to as amyloid precursor-like protein 1 (APL-1) or amyloid beta precursor protein-like 1 (APPL-1) [47].

Low identity values between *Drosophila melanogaster* and *Takifugu rubripes* (27.98%) highlight more distant evolutionary connections and possible differences in APP functions between these organisms, which may be related to adaptations to different ecosystems and physiological needs. However, the identity of around 32% in invertebrates points to some homologous aspects of APP proteins across these species.

We also conducted biomodeling of the interactions of H_2_S, HS^−^, and S_2_O_3_^2−^ with iNOS and the E2 domain of the APP protein. Docking results showed that S_2_O_3_^2−^ has the highest affinity for iNOS among the ligands studied, suggesting its potential effectiveness in inhibiting the enzyme or regulating its activity. In contrast, H_2_S and HS^−^ show weak affinities, consistent with their known roles as gaseous mediators with transient interactions with enzymes [3]. The binding of H_2_S and HS^−^ to iNOS revealed common key residues such as GLU 371 and ASP 376, suggesting similar interaction mechanisms. However, HS^−^ exhibits a unique and significant interaction with TYR 485, which may indicate differences in ligand stabilization in the active site. This difference in the interaction structure between the two ligands may explain why HS^−^, with its negative charge, tends to form stronger bonds with certain protein residues, despite its overall affinity being lower than that of S_2_O_3_^2−^.

The high affinity of S_2_O_3_^2−^ for iNOS is an important result and suggests that this ligand may exhibit more stable and prolonged interactions with the protein. Hydrogen bonding data for S_2_O_3_^2−^ may further explain its ability to form stable complexes with iNOS, making it a potential candidate for further investigation as a regulator of iNOS function. S_2_O_3_^2−^ may interact with key active site amino acids, thereby affecting the protein’s catalytic activity. It is known that S_2_O_3_^2−^ has a cytoprotective effect through interactions with pro-apoptotic proteins [21].

The E2 domain of APP plays an important role in its functionality, as it is involved in intermolecular interactions related to cell adhesion and signaling processes [49]. Key amino acid residues of this domain include those involved in hydrophobic and hydrogen bonding, as well as coordination with various ligand molecules. Key residues of the domain include histidine and cysteine, which may participate in metal coordination or interaction with small molecules [50].

H_2_S showed weak binding to the E2 domain, consistent with its general characteristics as a small molecule capable of diffusing across membranes and being rapidly metabolized within cells. Given H_2_S’s small size and low polarity [3], its binding to the E2 domain was limited by hydrogen bonds and weak van der Waals interactions. Key interactions of H_2_S with domain residues include weak contacts with amino acids such as ASP and LYS, likely due to H_2_S’s small size and low reactivity. These data suggest that H_2_S may slightly modulate E2 domain function, but its influence will mainly depend on concentration and local factors within the cellular environment. H_2_S was shown to form a significant number of hydrogen bonds with the E2 domain of APP. Key residues involved in these interactions include ASP 485, HIS 488, THR 489, MET 439, and GLN 510. These interactions indicate that H_2_S predominantly interacts with polar and charged amino acid residues, characteristic of weak hydrophobic interactions with additional hydrogen bonds.

Similarly, HS^−^ forms hydrogen bonds with residues such as GLU 443, ASP 485, HIS 514, THR 489, and ASN 429. These data confirm the high degree of similarity between the interactions of HS^−^ and H_2_S, although HS^−^ tends to form stronger hydrogen bonds due to its negative charge. In contrast to H_2_S and HS^−^, S_2_O_3_^2−^ forms an exceptionally large number of hydrogen bonds, predominantly with residues THR 489 and HIS 488. However, in many cases, the absence of hydrogen atoms may indicate the involvement of more complex coordination interactions that support complex stability. These interactions may explain the higher affinity values for S_2_O_3_^2−^, especially due to the presence of multiple coordination sites for binding with positively charged and polar residues.

Unlike H_2_S and HS^−^, S_2_O_3_^2−^ forms an exceptionally large number of hydrogen bonds, primarily with THR 489 and HIS 488 residues. However, in many cases, the absence of hydrogen atoms may indicate the involvement of more complex coordination interactions, which contribute to the stability of the complexes. These interactions may explain the higher affinity values of S_2_O_3_^2−^, particularly due to the presence of several coordination binding sites with positively charged and polar residues.

Docking and hydrogen bond analysis between H_2_S, HS^−^, and S_2_O_3_^2−^ with the E2 domain of the APP protein revealed that S_2_O_3_^2−^ shows the highest propensity to interact with the protein due to its higher affinity and greater number of interactions with positively charged or polar residues. H_2_S and its anion HS^−^ form weaker complexes with the protein, although HS^−^ tends to form stronger hydrogen bonds compared to neutral H_2_S. These findings provide important directions for further research into the role of these compounds in the context of APP interactions in both normal and pathological conditions.

Thus, we have thoroughly investigated the role of H_2_S in the expression and localization of iNOS and APP in neurons and glial cells of vertebrate and invertebrate animals following mechanical nervous tissue injury. We demonstrated that the levels of these proteins increase in damaged nerve and glial cells 24 h and 7 days after TBI. Seven days after injury, iNOS expression was detected not only in the cytoplasm but also in the nucleoplasm of damaged neurons. Additionally, iNOS was identified in axons. However, APP was exclusively localized in the cytoplasm of nerve cells and axons, and its level was induced by TBI. The use of Na_2_S led to a decrease in iNOS and APP levels in neural tissue cells after TBI, while the opposite effect was observed with AOAA administration.

For further detailed analysis, we studied the localization and expression of these proteins in the MPN of the crayfish *Astacus leptodactylus* following axotomy. As a result, we found that iNOS and APP were mainly localized in the cytoplasm of neurons, axons, and muscle tissue, with significantly less presence in glial cells 8 h after axon transection. A similar pattern was observed for APP. iNOS and APP were also detected in the total fraction of axotomized VNC ganglia. A neuroprotective effect was achieved using Na_2_S, which was counteracted when AOAA was added to the solution used to maintain the viability of the cold-blooded animals.

Additionally, we performed multiple sequence alignments of the amino acid sequences of iNOS and APP across evolutionarily diverse organisms. This study revealed a high degree of identity between the amino acid residues of iNOS and APP across various species of vertebrates and invertebrates. The highest identity was observed between humans (*Homo sapiens*) and mice (*Mus musculus*) for iNOS (81.12%) and between *Takifugu rubripes*, *Mus musculus*, and *Homo sapiens* for APP (over 69%). This indicates significant conservation of these proteins among mammals and shared evolutionary origins. However, in invertebrates such as *Drosophila melanogaster* and *Penaeus chinensis*, the identity was 44.56% and 49.34% for iNOS and 32% for APP, indicating differences in the structure and functions of these proteins across different groups of organisms.

The final step of our study involved biomodeling the interactions of H_2_S, HS^−^, and S_2_O_3_^2−^ with iNOS and the E2 domain of APP. It was shown that the binding of H_2_S and HS^−^ to iNOS reveals common key residues, such as GLU 371 and ASP 376, indicating similar interaction mechanisms. However, HS^−^ exhibits a unique and significant interaction with TYR 485, explaining the differences in ligand stabilization in the active site. The high affinity of S_2_O_3_^2−^ for iNOS makes it a potentially important candidate for further study as a regulator of iNOS function. S_2_O_3_^2−^ can interact with key amino acids of the active site, influencing the catalytic activity of the protein. On the other hand, H_2_S weakly binds to the E2 domain, primarily interacting with polar and charged amino acid residues. HS^−^ and S_2_O_3_^2−^ behave similarly, although S_2_O_3_^2−^ exhibits the greatest propensity for interaction with the protein due to its higher affinity and greater number of interactions with positively charged or polar residues.

## 4. Materials and Methods

### 4.1. Animals and Ethical Approval

The TBI study was conducted on adult male CD-1 mice, aged 14 to 15 weeks, with a body weight of 20 to 25 g. The mice were housed in specialized rooms in groups of six per cage at a temperature of 22–25 °C and an air exchange rate of 18 times per hour. Food and water were provided ad libitum.

The axotomy experiments were performed on crayfish (*Astacus leptodactylus)* caught from the Don River. The animals were kept in special aquariums filled with water under standard conditions. For housing, *Astacus leptodactylus* crayfish, 100 L aquariums with a closed-water circulation system were used. The water temperature was maintained at 18–20 °C. The pH level was kept within the range of 7.2–7.5, corresponding to a neutral or slightly alkaline environment, which is preferred by crayfish. Filtration and aeration were provided by an external filter with porous filling, ensuring continuous filtration and oxygenation of the water, preventing contamination, and maintaining optimal oxygen levels. A 12 h light cycle (12 h light, 12 h dark) was set to support the animals’ natural circadian rhythms. Water replacement (20% of the total volume) was performed weekly to prevent the accumulation of metabolites and other harmful substances. These conditions were chosen to create an environment as close to natural as possible, ensuring optimal conditions and minimal stress for the crayfish.

The studies complied with European Community Council Directive 86/609/EEC, Russian animal use regulations, GOST 33215-2014 (https://rm.coe.int/168007a6a8; accessed on 10 October 2017), and Protocol No. 2 of the DGTU Bioethics Commission. The animals were selected randomly, without consideration of external characteristics or behavior.

### 4.2. Objects and Procedure

TBI was modeled according to a standard protocol that was further modified. All mice were anesthetized with an intraperitoneal injection of chloral hydrate (300 mg/kg) prior to TBI modeling. The animals were then placed in a special system for TBI modeling. The mice’s heads were fixed, and foam was placed under the lower jaw to avoid fractures. A metal rod with a 3 mm diameter tip, 5 mm in length, and weighing 200 g was dropped from a height of 3 cm onto the non-exposed skull. The coordinates of the impact were set as 2 mm dorsal to the bregma and 1 mm lateral to the midline [51,52,53]. After recovery from anesthesia, the mice were returned to their normal housing. For this study, the animals were divided into two groups: a control group and an experimental group.

To investigate the role of H_2_S in regulating iNOS and APP expression and localization, the following modulators were used: H_2_S donor sodium sulfide (Na_2_S, 0.1 mg/kg; Khimikon, Rostov-on-Don, Russia) [13] or the CBS inhibitor aminooxyacetic acid (AOAA, 5 mg/kg; Tianjin Xidian Chemical Technology Co., Ltd., Tianjin, China) [15], which were administered intraperitoneally after TBI and daily until decapitation. The control group of mice received saline.

Neurotrauma in invertebrate animals involved axotomy on two neuroglial preparations of the crayfish *Astacus leptodactylus*. The first preparation was a stretch receptor of the crayfish (SRP), consisting of two mechanoreceptor neurons (MRNs) surrounded by satellite glial cells. The MRN dendrites were located on receptor muscles [54]. The SRP was isolated according to the method of Florey and Florey [55], which led to the axotomy of the neurons. In this procedure, the ventral carapace was attached to deep muscles, and the VNC was removed. Meanwhile, the dorsal carapace was cut along the midline, resulting in two symmetrical fragments, each containing five axotomized MRNs. These fragments were then mounted on a wax base in Petri dishes filled with van Harreveld’s physiological solution (in mM: NaCl-205; KCl-5.4; NaHCO_3_-0.24; MgCl_2_-5.4; CaCl_2_-13.5; pH 7.2–7.4), optimized for cold-blooded animals at 22–24 °C. Under stereomicroscopic control at 16× magnification, the remaining deep muscles overlying the sensory neurons were carefully removed. The SRP was excised with small segments of the chitinous carapace to which they were attached. The preparations were then placed in a special chamber filled with van Harreveld’s solution.

The second preparation was the VNC ganglia of the crayfish, consisting of six ganglia containing 500–1000 neurons connected by connectives made up of several hundred axons [51,56,57]. Following sectioning, this resulted in six bilaterally axotomized ganglia of the crayfish VNC.

Axotomy and isolation of ganglia were performed under stereomicroscopic control at 16–20× magnification. After removing the chitin from the ventral side of the crayfish abdomen, the VNC was quickly extracted using forceps and transferred to a chamber with van Harreveld’s solution. Control VNCs were cut at the anterior and posterior ends using sharp ophthalmic scissors, while experimental connectives were cut along the edges and between ganglia. This resulted in six bilaterally axotomized ganglia. The axotomized ganglia were then placed in chambers with van Harreveld’s solution and incubated for 8 h post-axotomy at 22–24 °C. For the axotomy study, the following H_2_S modulators were used at final concentrations: Na_2_S (250 µM) and AOAA (3 mM). These were added to the chamber containing van Harreveld’s solution, where the neuroglial preparations of invertebrates were kept.

### 4.3. Immunofluorescent Microscopy

To determine the localization of iNOS and APP in the brain 24 h and 7 days after TBI, the following protocol was used. The area around the necrotic focus caused by the weight strike on the skull, as well as the left, undamaged hemisphere, was extracted. This study area was the parietal cortex of the brain. The excised brain cortex section was fixed for 6 h in 4% paraformaldehyde and then placed in 4% agarose gel (low-melting agarose, Sigma Aldrich, Burlington, MA, USA).

Sections of agarose blocks approximately 20 µm thick were obtained using a Leica VT 1000 S vibratome (Leica Biosystems, Nussloch, Germany). After PBS washing, the sections were incubated for 1 h at room temperature in a blocking solution consisting of 5% BSA and 0.3% Triton X-100. The sections were then incubated with rabbit primary antibody against iNOS (1:100, PAA837Mu03, Cloud-Clone, Wuhan, China) or APP (1:100, SAB4200536, Sigma Aldrich), recognizing the N-terminal part of APP, and mouse antibody against the neuron-specific nuclear protein NeuN (1:1000; FNab10266, FineTest, Wuhan, China), or Nse (1:1000, SAB4200571, Sigma Aldrich), or the astrocyte marker GFAP (1:1000; SAB4200571, Sigma Aldrich) for two days at 4 °C. After washing with PBS, the sections were incubated with secondary antibodies anti-rabbit IgG (H + L) Fluor488-conjugated (1:500; S0018, Affinity Biosciences, Zhenjiang, China) and anti-mouse IgG (H + L) Fluor594-conjugated (1:500; S0005, Affinity Biosciences, Zhenjiang, China). Negative control involved the absence of primary antibodies. Hoechst 33342 (40 µM; 10 min) was used to visualize the nuclei of neurons and glial cells. The sections were placed in 60% glycerol and examined using a fluorescent microscope, Altami LUM 1 (Ningbo Haishu Honyu Opto-Electro Co., LTD., China, together with Altami, Russia), equipped with a high-resolution digital camera (EXCCD01400KPA, Hangzhou ToupTek Photonics Co., Ltd., Hangzhou, China). Each group included 6 animals.

Colocalization of iNOS and APP with the neuron marker NeuN, GFAP, or Nse was evaluated using Image J version 1.51r (http://rsb.info.nih.gov/ij/, accessed on 10 February 2017) software with the JACoP plugin [58]. The colocalization coefficient M1 reflects the proportion of pixels containing both green (iNOS and APP) and red signals (NeuN or GFAP, Nse) relative to the total signal recorded in the green channel [59]. Calculations were based on at least 100 cells. To quantify the average fluorescence level of iNOS and APP in experimental and control samples, 10 control and 10 experimental images for each of the 6 mice were used. The average (area-based) fluorescence of the cytoplasm and nucleus for each cell was assessed, and the values were averaged or normalized to the background fluorescence intensity using the following formula:

$$I_{norm}=\frac{I_{meas}-I_{back}}{I_{back}}$$

where *I*_meas_ is the average intensity in the studied area, and *I*_back_ is the average background intensity.

Eight hours after axotomy of MRNs, immunofluorescent analysis of iNOS or APP localization was conducted. The same primary antibodies were used for the TBI model. Secondary antibodies Fluor488-conjugated (1:500; S0018, Affinity Biosciences, China) were applied in a 1:500 dilution in PBS. The neuroglial MRN preparations were fixed for 24 h in 4% paraformaldehyde solution in PBS. They were then washed for 24 h in a mixture of 1% bovine serum albumin (BSA), 0.2% NaN3, and 1% Triton X-100. Following this, the samples were incubated for 24 h in the primary antibody solution, washed, incubated for 24 h in the secondary antibody solution, and washed again for 24 h at +4 °C.

Hoechst-33342 (SB-G1127-1ML, Servicebio Technology, Ltd., Wuhan, China) (40 µM) was used for 10 min at +25 °C to visualize cell nuclei in the CSR. The preparations were mounted on a glass slide in a drop of glycerol and covered with a coverslip. The samples were then photographed using a fluorescence microscope. The level of fluorescence of antibodies to target proteins was assessed in different elements of the CSR: the nucleus and cytoplasm of the MRN, axons, dendrites, and glia. The calculations were performed similarly to those for TBI.

### 4.4. Western Blot

The expression of iNOS and APP in brain tissue after TBI and in axotomized VNC ganglia was studied using the Western blot method. To achieve this, 24 h and 7 days after TBI, mice were sacrificed by decapitation, the brain was isolated on ice, and the area corresponding to the penumbra was excised. A similar region was excised from the contralateral cortex of the same mouse. The samples were then homogenized on ice for 5 min with the addition of a lysis buffer (IS007 Lysis Buffer-3, Cloud-Clone Corp., Wuhan, China), supplemented with a cocktail of protease and phosphatase inhibitors (PPC1010, Sigma-Aldrich) and benzonase nuclease (E1014, Sigma-Aldrich). The samples were subsequently centrifuged for 20 min at 10,000–11,000× *g* at 4 °C in a Hanil Scientific M15R centrifuge with a cooling system (Hanil Scientific Inc., Kimpo, Republic of Korea). The resulting supernatant was collected for further analysis.

VNC ganglia after bilateral axotomy were incubated for 8 h in Van Harreveld’s solution with or without H_2_S modulators at room temperature. For sufficient biological material, ganglia from five experimental and control VNCs were combined. Sample preparation was carried out in the same way as described above. The protein content was determined using the Bradford reagent (K002-500, FineTest, China) on a SpectroSTAR Nano Plate Reader spectrophotometer (BMG Labtech, Ortenberg, Germany). The determination of protein concentration in samples was carried out in three repetitions, using the average value in the calculation. The protein concentration in the samples ranged from 4 to 6 mg/mL.

Samples containing 10–20 µg of protein in 15 µL were subjected to electrophoretic separation in a 10% polyacrylamide gel in the presence of sodium dodecyl sulfate using a mini-PROTEAN Tetra cell (Bio-Rad, Hercules, CA, USA). Affinity Prestained Protein Ladder (KF8009, Affinity, Shanghai, China) was used as a standard protein marker. After separation, the proteins were transferred onto a PVDF membrane (polyvinyl difluoride membrane 162-0177, Bio-Rad) using the Trans-Blot^®^ Turbo Transfer System (Bio-Rad, USA). The washed membrane was incubated for 1 h in a blocking buffer (TBS 1% Casein Blocker, Bio-Rad) and then overnight at 4 °C with primary rabbit antibodies against iNOS (1:100, PAA837Mu03, Cloud-Clone, China), APP (1:100, SAB4200536, Sigma Aldrich), and β-actin (1:1000; PAB340Mi01, Cloud-Clone Corp., China). Membranes were then washed in Tris-buffer with 0.1% Tween-20 (TTVS, 10 mM; pH 8) and incubated for 1 h at room temperature with secondary anti-rabbit IgG peroxidase antibodies (1:1000; S0001, Affinity Biosciences, China).

Protein detection was performed using the KF8001 Affinity™ ECL kit for chemiluminescent detection of HRP conjugates (KF8001, Affinity Biosciences, China). Chemiluminescence was analyzed using the high-resolution gel documentation system SH-Advance523 (Shenhua Science Technology Co., Ltd. (SHST), Hangzhou, China). The obtained data were normalized to β-actin. This makes up 10–20% of the total cellular protein and is considered an evolutionarily conserved protein. The resulting images were processed using the SHST Analysis software package.

### 4.5. Bioinformatics Analysis

The Clustal Omega method was used to perform multiple sequence alignments of amino acid sequences, available through the web interface of the European Bioinformatics Institute (EBI) (www.ebi.ac.uk/Tools/msa/clustalo; accessed on 10 October 2023) [60]. This method is based on a progressive alignment algorithm and is optimized for accurate and scalable analysis of large datasets. Amino acid sequences were obtained using the National Center for Biotechnology Information (NCBI) resource (www.ncbi.nlm.nih.gov; accessed on 10 October 2023), a central portal for bioinformatics data. The protein database, which includes protein sequences from various species, was used for the search. Sequences were selected based on their annotations and relevance to this study. The alignment was carried out using the corresponding tool on EBI, accessible via the “Launch Clustal Omega” link. Standard Clustal Omega parameters were applied to ensure high alignment accuracy. This method helped identify conserved and variable regions in the amino acid sequences, which is important for further analysis of the evolutionary and functional aspects of proteins.

After the multiple sequence alignment was completed, an identity matrix was generated—a table in which the percentage identity for each pair of sequences was calculated. The identity reflects the proportion of identical amino acids in the aligned sequences. This is a critical indicator for assessing the degree of evolutionary relatedness between sequences. The identity matrix was created based on alignment results for all pairs of proteins used in this study. Each element of the matrix represents the percentage of amino acid matches for the corresponding pair of sequences. High identity values (greater than 30%) suggest possible protein homology, confirming their common evolutionary origin. Low values (less than 25%) may indicate a distant evolutionary relationship or functional divergence. The matrix allows for the visualization of evolutionary relationships between proteins from different organisms, highlighting conserved regions and potential functional changes. The analysis was performed on APP and iNOS proteins across different species and classes (Table 5).

The resulting multiple alignments were analyzed to identify conserved domains relevant for functional and structural studies and to construct phylogenetic trees to analyze the evolutionary history of proteins.

### 4.6. Biomodeling of H_2_S, HS^−^, and S_2_O_3_^2−^ Interaction with iNOS and APP

The crystal structures of iNOS and APP obtained from the RCSB Protein Data Bank (PDB ID: 1DWW for iNOS and PDB ID: 3NYL for the E2 domain of the APP protein [73]) were used for modeling. The structures were downloaded in PDB format and used for subsequent molecular docking. Hydrogen sulfide molecule H_2_S, HS^−^, and S_2_O_3_^2−^.

Molecular docking was performed using the SwissDock web service (http://www.swissdock.ch/; accessed on 10 October 2024). The structures of proteins (iNOS and APP) and ligands (H_2_S, HS^−^, and S_2_O_3_^2−^) were loaded into the corresponding fields on SwissDock [74,75]. The default settings of the service were used, including the flexible ligand docking mode and a local search area around the active sites of proteins. The docking results are presented as protein–ligand complex conformations with an estimate of the binding energy (ΔG).

The obtained protein–ligand complex conformations were analyzed using UCSF Chimera (version 1.15). PDB files with the docking results were loaded into Chimera for visualization and analysis of interactions between the protein and ligand.

Visualization of the protein–ligand complex was accomplished using 3D rendering in UCSF Chimera, including display of protein secondary structure, H_2_S, HS^−^, and S_2_O_3_^2−^ molecules, and surface interactions.

### 4.7. The Statistical Analysis

One-way analysis of variance (ANOVA) with Dunnett’s post hoc test was used for statistical analysis of the data. The Shapiro–Wilk and Brown–Forsythe tests were used to check the normality of distribution and homogeneity of variances, respectively. In cases of non-compliance with these criteria, the non-parametric Kruskal–Wallis H test was used. All analyses were performed blindly. Differences were considered significant at *p* < 0.05 and *n* = 6. Results are presented as mean values ± standard errors of the means.

## Figures and Tables

**Table 1:** Percent identity matrix for iNOS—generated by Clustal2.1.

1	*Oncorhynchus mykiss*	100.00	60.17	58.18	58.18	43.19	46.02
2	*Gallus gallus*	60.17	100.00	67.05	70.48	44.57	47.68
3	*Mus musculus*	58.18	67.05	100.00	81.12	44.56	48.21
4	*Homo sapiens*	59.52	70.48	81.12	100.00	44.56	49.34
5	*Drosophila melanogaster*	43.19	44.57	44.56	44.56	100.00	56.98
6	*Penaeus chinensis*	46.02	47.68	48.21	49.34	56.98	100.00

**Table 2:** Percentage identity matrix for APP–generated by Clustal2.1.

1	*Takifugu rubripes*	100.00	69.43	69.43	31.69	27.98	32.21
2	*Mus musculus*	69.43	100.00	96.62	32.38	27.47	30.87
3	*Homo sapiens*	69.43	96.62	100.00	32.03	27.34	30.71
4	*Doryteuthis pealeii*	31.69	32.38	32.03	100.00	31.32	33.33
5	*Drosophila melanogaster*	27.98	27.47	27.34	31.32	100.00	45.88
6	*Hirondellea gigas*	32.21	30.87	30.71	33.33	45.88	100.00

**Table 3:** H_2_S, HS^−^, and S_2_O_3_^2−^ poses ranked by their docking score with the iNOS monomer in the docking models.

	Models		
	H_2_S	HS^−^	S_2_O_3_^2−^
	Calculated Affinity (kcal/mol)		
1	−0.785	−0.785	−3.465
2	−0.774	−0.774	−3.360
3	−0.759	−0.742	−3.354
4	−0.742	−0.723	−3.255
5	−0.723	−0.694	−3.252
6	−0.694	−0.626	−3.240
7	−0.669	−0.553	−3.170
8	−0.623	−0.513	−3.125
9	−0.577	−0.505	−3.123
10	−0.525	−0.497	−3.093
11	−0.513	−0.493	−3.089
12	−0.511	−0.489	−3.063
13	−0.500	−0.485	−2.972
14	−0.487	−0.480	−2.897
15	−0.484	−0.474	−2.840
16	−0.474	−0.470	−2.765
17	−0.470	−0.465	−2.721
18	−0.459	−0.458	−2.696
19	−0.439	−0.457	−2.685
20	−0.438	−0.443	−2.666

**Table 4:** H_2_S, HS^−^, and HS_2_O_3_^2−^ poses ranked by their docking scores with the E2 domain of APP in docking models.

	Models		
	H_2_S	HS^−^	S_2_O_3_^2−^
	Calculated Affinity (kcal/mol)		
1	−0.701	−0.700	−2.636
2	−0.647	−0.651	−2.575
3	−0.600	−0.594	−2.486
4	−0.591	−0.585	−2.483
5	−0.581	−0.581	−2.390
6	−0.570	−0.570	−2.323
7	−0.528	−0.528	−2.226
8	−0.528	−0.527	−2.199
9	−0.527	−0.516	−2.193
10	−0.520	−0.510	−2.132
11	−0.517	−0.481	−2.119
12	−0.481	−0.474	−2.088
13	−0.456	−0.474	−1.943
14	−0.453	−0.473	−1.925
15	−0.439	−0.430	−1.911
16	−0.424	−0.424	−1.867
17	−0.398	−0.418	−1.850
18	−0.396	−0.416	−1.757
19	−0.391	−0.410	−1.746
20	-	−0.390	−1.745

**Table 5:** Amino acid sequences of iNOS and APP used for multiple alignments from different organisms to assess the evolutionary conservation of these proteins.

iNOS			
Class	Animal Species	Sequence Identifier	Number of Amino Acid Residues
Insecta	*Drosophila melanogaster*	Accession: Q27571.3 GI: 190358925 [61]	1349
Malacostraca	*Penaeus chinensis*	Accession: AFJ74715.1 GI: 387308611 [62]	1193
Actinopterygii	*Oncorhynchus mykiss*	Accession: NP_001117831.1 GI: 185133861 [63]	1083
Aves	*Gallus gallus*	Accession: NP_990292.2 GI: 2125314497 [64]	1136
Mammalia	*Mus musculus*	Accession: NP_035057.1 GI: 6754872 [65]	1144
Mammalia	*Homo sapiens*	Accession: AAI44127.1 GI: 219520412 [66]	1153
**APP**			
Insecta	*Drosophila melanogaster*	Accession: NP_476626.2 GI: 24638892 [67]	887
Cephalopoda	*Doryteuthis pealeii*	Accession: ABI84193.2 GI: 116520773 [68]	612
Malacostraca	*Hirondellea gigas*	Accession: LAB65993.1 GI: 1371970032 [69]	753
Actinopterygii	*Takifugu rubripes*	Accession: AAD13392.1 GI: 4204468 [70]	737
Mammalia	*Mus musculus*	Accession: NP_001185752.1 GI: 311893401 [71]	770
Mammalia	*Homo sapiens*	Accession: NP_000475.1 GI: 4502167 [72]	770

## Data Availability

The original contributions presented in this study are included in the article/Supplementary Materials. Further inquiries can be directed to the corresponding author.

## Supplementary Materials

The following supporting information can be downloaded at: https://www.mdpi.com/article/10.3390/ijms252211892/s1.
